# The evolution of facility-based deliveries at primary healthcare centres during an insecurity and conflict crisis in Burkina Faso: a geospatial analysis

**DOI:** 10.1186/s13031-025-00723-8

**Published:** 2025-11-03

**Authors:** Felix Amberg, Manuela De Allegri, Valéry Ridde, Ali Sie, Mariam Seynou, Kadidiatou Kadio, Sayouba Dianda, Julia Lohmann, Karl Blanchet, Neha S. Singh, Emmanuel Bonnet

**Affiliations:** 1 Heidelberg Institute of Global Health, Heidelberg University Hospital and Medical Faculty, Heidelberg University, Heidelberg, Germany; 2 Institut de Recherche pour le Développement, Centre Population et Développement, Université Paris Cité, Paris, France; 3 Centre de Recherche en Santé de Nouna, Nouna, Burkina Faso; 4 Institut de Recherche en Science de la Santé, Ouagadougou, Burkina Faso; 5 University of Bonn, Bonn, Germany; 6 Institute for Medical Information Processing, Biometry, and Epidemiology, Ludwig Maximilian University of Munich, Munich, Germany; 7 Geneva Centre of Humanitarian Studies, Faculty of Medicine, University of Geneva, Geneva, Switzerland; 8 Department of Global Health and Development, Faculty of Public Health and Policy, London School of Hygiene & Tropical Medicine, London, UK; 9 Institut de Recherche pour leDéveloppement, Centre National de la Recherche Scientifique, Université Paris 1 Panthéon-Sorbonne, Paris, France

## Abstract

**Background:**

Access to healthcare in Burkina Faso, particularly obstetric care, is severely reduced by nearby armed conflict. However, systematic assessments of the spatial distribution of facility-based delivery rates and their evolution, as well as how they relate to conflict intensity, facility characteristics, and geo-spatial determinants, are absent.

**Methods:**

We analysed spatial and temporal shifts in facility-based deliveries in Burkina Faso’s primary healthcare centers in relation to conflict. Using spatial analyses, we examined how conflict-related deaths influenced delivery patterns, considering variations by facility type and pre-conflict service volume. We obtained monthly healthcare facility data (2016–2021) from Burkina Faso’s Health Management Information System (HMIS) and conflict event data from the Uppsala Conflict Data Program (UCDP). The study covered all primary healthcare centers in conflict-affected northern and eastern districts (> 10 UCDP conflict deaths, 2016–2021) and neighboring southern districts: 854 CSPSs (Centres de Santé et de Promotion Sociale) and 53 CMs (Centres Médicaux).

**Results:**

The study identified geographical variations in facility-based delivery rates, notably from 2018–2019, with spatial clusters of lower rates becoming predominant in northern areas and higher rates doubling along southern routes and cities. This shift coincided spatially and timely with conflict escalation. In conflict hotspots, the average monthly rate of facility-based deliveries decreased over time, irrespective of pre-conflict service volume. However, CM facilities showed an upward trend, contrasting CSPS facilities. Outside conflict hotspots, facilities with exceptional pre-conflict service volume showed similar upward trends, while low- and high-volume facilities showed moderate increases. CM consistently maintained higher facility-based delivery rates over time than CSPS facilities.

**Conclusion:**

This research provides crucial insights for strengthening Burkina Faso’s health system resilience to conflict by spatially identifying how facility characteristics and geo-spatial determinants shape healthcare disruptions. Mapping these elements at a fine scale enables adaptive policy interventions and dynamic resource allocation based on evolving conflict dynamics, enhancing obstetric care during conflict. By integrating the applied geospatial methods into national health systems, we can enhance responsiveness, enabling targeted and timely interventions, as well as efficient and flexible resource distribution (e.g., funding, personnel, and medical supplies). This also supports improved healthcare demand forecasting, ultimately ensuring a more proactive, data-driven, and conflict-sensitive approach to maternal health policy planning in crisis response.

**Supplementary Information:**

The online version contains Supplementary material available at 10.1186/s13031-025-00723-8.

## Introduction

Since 2012, in sub-Saharan Africa and the Sahel region in particular, armed conflicts have had profound effects on healthcare systems, disrupting access to care and straining human and financial resources. [1–3] Armed conflicts instil a widespread feeling of insecurity among both the population and healthcare workers in primary healthcare centres, bearing the potential to disrupt the operation and effectiveness of healthcare centres, thereby exacerbating barriers to accessing healthcare [4]. These disruptions significantly contribute to a low prevalence of facility-based deliveries and consequently to elevated maternal mortality rates. [5–7].

In Burkina Faso, evidence suggests that armed conflict affects the organization of healthcare services and decreases access to both maternal and child health services [8–10]. The presence of terrorist groups confronting the government and exacerbating ethnic and social divisions is fostering a pervasive sense of insecurity. This environment poses heightened risks for women and children seeking essential care, as well as for the dedicated local health workers trying to deliver it [1]. Consequently, access to healthcare has become constrained, while the capacity of local health systems to deliver adequate services has been compromised. [11, 12].

In a previous study, we assessed the impact of nearby armed conflict events (within 25 km of primary healthcare centres) on access to six different essential maternal and child health services using negative binomial regression models with healthcare facility fixed effects. [10] Our regression analysis showed that access to health services in Burkina Faso’s primary healthcare facilities is profoundly affected by nearby conflict, but services of a different nature are affected differently, depending on the intensity and duration of the conflict as well as facility type and location. For example, we observed that the adverse effects of armed conflict on access to healthcare at primary healthcare facilities are more pronounced in rural compared to urban settings.

Despite these insights, the understanding of the exact spatial distribution of facility-based delivery rates and their evolution remains limited in Burkina Faso. Furthermore, the relationship between potential spatial shifts and various factors—such as facility characteristics and geo-spatial determinants—have not been thoroughly examined. In this study, we use the term *geo-spatial determinants* to refer to factors related to the spatial positioning of primary healthcare facilities that can explain potential spatial shifts in the access to obstetric care. These are either directly modelled in our analyses or considered in the interpretation of the results and include proximity to armed conflict events and conflict-related deaths, location relative to major roads and (semi-)urban or rural settings, the surrounding target population, and spatial clustering of facilities. A few studies conducted in other Sahelian countries affected by conflict exist, but, except for a work by Bonnet and colleagues in Mali [13], their analyses do not provide insight into the matter we examined in this research. [14–17].

Specifically, with this work, we aimed at assessing how facility-based delivery rates developed spatially over time in relation to the location and intensity of conflict. In light of the abovementioned knowledge gaps, we also aimed at examining how the combination of facility characteristics and geo-spatial determinants shaped the evolution of the observed patterns. In order to fulfil this overarching aim, we built on the methodological approach used by Bonnet and colleagues in Mali [13], and pursued three specific objectives: (1) to analyse the geographical dispersion of facility-based deliveries over time; (2) to examine the influence of nearby armed conflict on this evolution, and (3) to assess how structural facility characteristics such as the pre-conflict service volume and the type of the healthcare facility contributed to disparities in the effects of nearby armed conflicts on facility-based delivery, depending on the level of conflict exposure across geographical areas.

This research is significant as it yields great potential to promote resilience of the Burkina Faso’s health system to conflict shocks. In this context, we refer to resilience as a healthcare facility’s ability to proactively foresee, withstand, absorb, adapt to, and recover from the impacts of conflict. By mapping healthcare disruptions due to nearby conflict and identifying which facility characteristics and geo-spatial determinants shape a facility’s resilience to these conflict shocks over time, this study provides insights that can inform adaptive policy interventions and dynamic resource allocation strategies based on conflict dynamics, ultimately enhancing the capacity of healthcare facilities to sustain obstetric care in times of conflict.

## Methods

### Study area

Burkina Faso is a landlocked country with a population of approximately 21 million [18]. The country faces significant economic challenges, with over 40% of its population living on less than 420 FCFA (< 1 USD) per day [19]. It ranks 184th out of 191 countries on the 2021/22 Human Development Index, indicating its status as one of the world’s poorest countries [20]. Despite previously being considered a country of stability in a volatile region, marked by peaceful coexistence among diverse ethnic groups and progress toward democratization, Burkina Faso has faced turmoil since 2016.Terrorist groups, including Ansarul Islam, Jama’at Nusrat al-Islam wal Muslimeen (JNIM), and the Islamic State in the Greater Sahara (ISGS), have instigated violence primarily in the north, later spreading to the east [21]. This escalation, compounded by factors such as banditry, inter-communal conflicts, civil unrest, and political instability, has led to a humanitarian crisis characterised by mass displacement, food insecurity, and widespread human rights violations. Despite governmental efforts to address these challenges, the situation remains volatile and complex. In 2021, Burkina Faso recorded the second-highest number of terror-related deaths globally [22], and in 2022, it experienced two coup d’états, further impeding the government’s ability to combat the terrorist groups. [23].

### Data and data sources

Our research relied on two primary sources of secondary data: Burkina Faso’s national Health Management Information System (HMIS) and the Uppsala Conflict Data Program Georeferenced Events Dataset (UCDP GED).

The HMIS data comprises monthly counts of services provided by specific facilities in Burkina Faso [24]. These data have demonstrated sufficient quality for meaningful and reliable analyses [25–28]. For our study, we utilised HMIS data spanning from 2016 to 2021, coinciding with the onset of the security crisis in Burkina Faso in 2016. The conflict intensified further after 2021; however, since then, the quality of HMIS data in conflict-affected health districts has significantly declined. Considering the trade-off between extending the study period to include additional years with substantially lower quality data, we opted to restrict our analysis to HMIS data from 2016 to 2021. Additionally, cleaning the HMIS data and geocoding healthcare facilities from 2016 to 2021 presented significant challenges due to inconsistencies in facility naming and the lack of GPS information in the HMIS dataset. It is worth noting that within the 2016–2021 study period, the evolving conflict dynamics provided sufficient variation to analyse how spatial shifts in conflict influence the spatial distribution of facility-based deliveries.

We focused on primary healthcare centres(CSPS – Centres de Santé et de Promotion Sociale – and CMs – Centres Médicaux), which are largely located in rural and semi-urban areas and are therefore more directly affected by the predominantly rural conflict in Burkina Faso than secondary and tertiary health facilities [29]. The facility types CSPS and CM correspond to the first level in the healthcare pyramid and are essential for facility-based deliveries. They handle most routine deliveries, providing basic obstetric services and ensuring safe childbirth in community settings. When complications exceed their capacity, cases are referred to Centres Médicaux avec Antenne Chirurgicale (CMAs). CM and CSPS facilities differ in that in the CMs there is a doctor (MD), which is not the case in the CSPSs. Additionally, CMs have more personnel with higher qualifications and, ideally, access to a larger technical platform that allows for certain biological and radiological examinations, while CSPSs rely solely on nurses and midwives and typically handle lower patient volumes. However, many CMs are CSPSs that have evolved into CMs with the arrival of a doctor, but the evolution of a minimum of technical facilities to facilitate activities for which a doctor is responsible can be slow. As a result, they remain two primary consultation structures that, in practice, often do not differ considerably, except for the presence of a doctor, larger patient volume, and some biology tests. In some cases, CSPSs are even more frequented than CMs due to the distance of these CMs or the population covered or for other reasons such as the refusal of certain populations to go to rival village healthcare facilities unless they have no choice. CMAs are excluded from the analysis since they correspond to the second tier of the first level of care that complements the 1 st tier by providing a complementary package of activities. In addition, their inclusion would have complicated the calculation of the target population living near the facilities, which is detailed below, as the catchment areas overlap and are larger compared to CSPS and CMs. [30].

Except for a preparatory health district-level analysis conducted throughout the country to identify the study area, which is detailed in the next section, our focus was on facilities located within health districts that are located in the north and east and are most affected by conflict, i.e., > 10 conflict deaths from 2016 to 2021 (Djibo, Gorom-Gorom, Dori, and Sebba in the Sahel region; Thiou, Titao, and Ouahigouya in the Nord region; Kongoussi, Barsalogho, Tougouri, and Kaya in the Centre-Nord region; Gayeri, Fada N’Gourma, Diapaga, and Pama in the Est region; and Nouna and Tougan in the Boucle du Mouhoun region), and their immediately neighbouring districts to the south (Gourcy, Seguenega, and Yako in the Nord region; Boussouma and Boulsa in the Centre-Nord region; Mani and Bogande in the Est region; and Solenzo, Dedougou, and Toma in the Boucle du Mouton region). Zooming into the conflict-affected districts and their neighbouring districts to the south allowed us to better detect areas in which changes in the spatial heterogeneity of facility-based deliveries were particularly due to nearby conflict or displacement and to further examine the most interesting and deviant cases within these areas.

Geocoding of these facilities was facilitated by GPS information from the Health Resources Availability Mapping System (HeRAMS) dataset and the Base Nationale de Données Topographiques (BNDT), a comprehensive national locality database. After geocoding, our dataset included 864 CSPS and 53 CM facilities.

Data cleaning steps including outlier detection and imputation of missing values in the HMIS data are detailed in Supplementary material 1. Unfortunately, while data on the target population is available at the health district level in Burkina Faso, there is no such data at the facility level. Supplementary material 1 Fig. 1 and 2 illustrate how we defined the target population living around the facilities using a spatial technique, which is also detailed in Supplementary material 1. The rates are needed as the spatial analyses conducted in this paper require rates instead of pure counts.

The UCDP GED offers detailed information on conflict events, including time, location, type, and intensity, spanning from 1946 to the present day [31–33]. A conflict event in the UCDP GED is defined as “an incident where armed force was used by an organised actor against another organised actor, or against civilians, at a specific location and a specific date” [33]. The UCDP GED compiles information from various sources such as news reports, NGO documentation, case studies, and historical archives [34]. For our study, we focused on conflict events occurring in Burkina Faso from 2016 to 2021. During this period, the UCDP GED documented 673 conflict events in Burkina Faso, resulting in 4146 conflict-related deaths. Utilizing GPS information from these datasets enabled us to conduct geospatial analyses (Supplementary material 1 Fig. 1 and 2).

### Health district analysis

To identify our regional focus, we first conducted a descriptive, preparatory analysis of facility-based deliveries across the 70 health districts in Burkina Faso. We started by aggregating the number of CSPS and CM-based deliveries per health district and dividing it by the number of expected deliveries within each district (from annual HMIS statistics) before we could track the change of this rate from 2016 to 2021. Next, we regressed the monthly rate on the respective monthly number of UCDP conflict deaths within each district (any, below and above median, and quintile increases in UCDP conflict deaths for all conflict events in our dataset, plus one indicator for districts with more than 50 conflict deaths). This regression analysis at the health district level was conducted using monthly data from January 2013 to December 2021.

### Geographical analysis of the distribution of facility-based deliveries over time

To address objective 1, we progressed in stages, guided by the work of Bonnet and colleagues. [13].

#### Global autocorrelation analysis

**Fig. 1:**
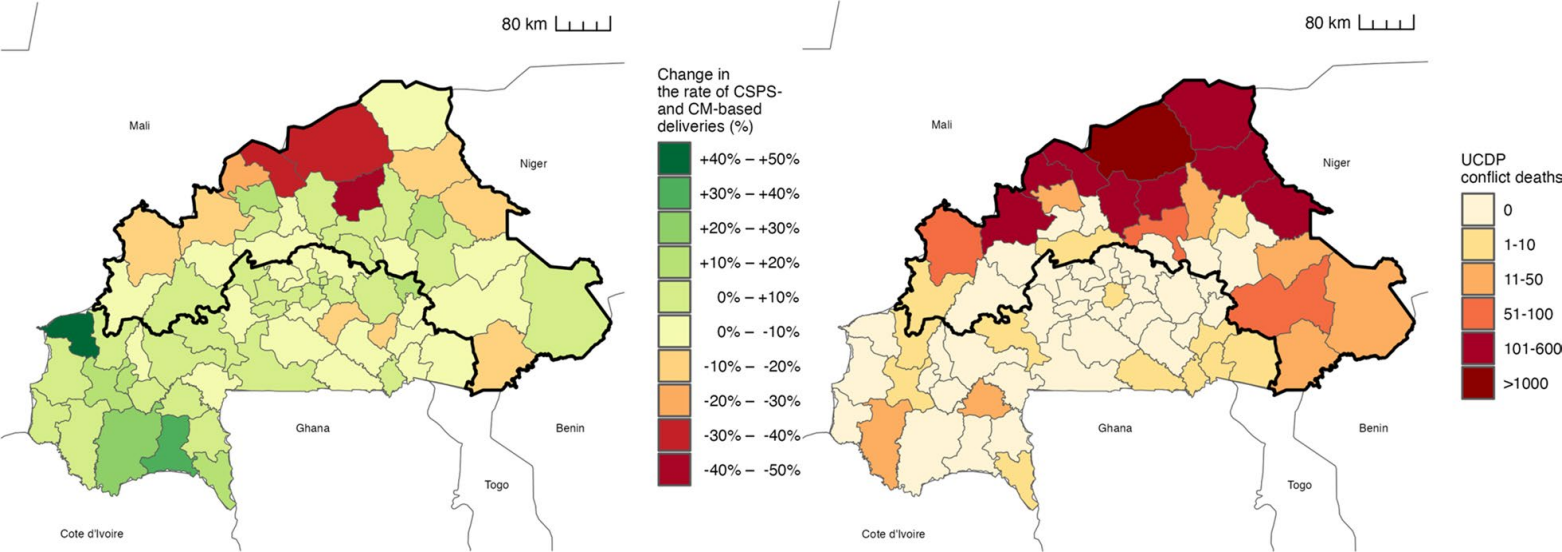
**Health district analyses: Change in the rate of facility-based deliveries (left) and sum of UCDP conflict deaths (right), by health district (2016–2021).** Percentage change in the rate of facility-based deliveries in CSPS and CM facilities between 2016 and 2021, by health district (left); and sum of UCDP conflict deaths between 2016 and 2021, by health district (right). The area examined in the geospatial analyses (see Fig. 2–4) is displayed with thick borders. The left panel illustrates that, from 2016 to 2021 and within the thick borders, the share of deliveries in CSPSs and CMs sharply declined in the northern health districts. For instance, Titao, Djibo, and Barsalogho experienced reductions ranging from 30 to 50 percent (highlighted in red). In contrast, the share of deliveries in the neighbouring health districts to the south increased by 0 to 20 percent (highlighted in green). The right panel reveals that the health district showing declines in panel A also experienced a high number of UCDP conflict deaths during the same period. For instance, Titao, Djibo, and Barsalogho experienced more than 100 conflict deaths between 2016 and 2021

First, for a more granular exploration of spatial and temporal patterns in deliveries at the level of individual primary healthcare facilities, we calculated a global spatial autocorrelation. Accessibility to healthcare varies within health districts, underscoring the importance of conducting localised analyses to avoid ecological fallacies that may obscure these disparities [35]. The global spatial autocorrelation aimed at ascertaining whether there exists a correlation between the number of deliveries in CSPSs and CMs and the spatial relationships among these primary healthcare centres. To account for neighbouring values, we utilised the Moran index [36]. This index computes the average of the products of normalised values of pairs of points, weighted by the distance between them:

$${I}_{Moran}= \frac{\sum_{i=1}^{n}\sum_{j=1}^{n} {w}_{ij}\left({x}_{i}-\overline{x }\right)({x}_{j}-\overline{x })}{\sum_{i=1}^{n}\sum_{j=1}^{n}{w}_{ij} \sum_{i=1}^{n}({x}_{i}-\overline{x }{)}^{2}}$$

where $i$, $j$ = spatial unit; $n$ = the number of spatial units; ${x}_{i}$ is the value of the variable in the unit $i$; $\overline{x }$ is the average of $x$; and ${w}_{ij}$ are the elements of the spatial interaction matrix to define spatial contiguity, distances or shared borders. This calculation was performed only within the zoomed-in area (depicted with thick borders in Fig. 1) for the reasons explained in the data section. A positive Moran index signifies heterogeneity within the study area, indicating the presence of clusters of primary healthcare facilities that are closely located and exhibit either lower or higher numbers of deliveries than expected under random spatial distribution. We identified the neighbours for the autocorrelation using two different approaches. First, we considered all neighbouring facilities within 25 km (d = 25). We used 25 km as a distance threshold because it approximates the maximum distance that could reasonably be travelled on foot within a day, making it a plausible proxy for healthcare access and suitable for the autocorrelation analysis. In addition, we used this value to maintain consistency with our previous study, which employed 25 km conflict exposure bands, thereby facilitating comparability. Second, we considered the 10 nearest neighbours only—the average number of health facilities within a 25 km radius in the study setting—to test robustness of the autocorrelation results (k = 10). We also conducted two additional robustness checks: one with lower thresholds (d = 10, k = 5) and another with higher thresholds (d = 50, k = 20).

The global autocorrelation was used to establish whether facility-based deliveries exhibited an overall spatial pattern, ensuring that clustering was not random. Conducting this step before a spatial scan (see below) provided justification for applying local cluster detection by confirming the presence of spatial dependence in the data.

#### Spatial scan analysis

Second, we employed spatial scan statistics to examine the spatial variability of facility-based deliveries in primary healthcare centres in the zoomed-in area [37]. This method involved covering the study area with circular scanning windows that vary in size, thereby identifying clusters with an unusually high or low concentration of facility-based deliveries using the log-likelihood ratio test, conducting Monte Carlo simulations to test significance, and reporting the statistically significant clusters for further investigation (groups of facilities that are geographically close and have a lower or higher rate than expected, i.e., if the rates were randomly or uniformly distributed over the study area). [38] When computing the spatial scan statistics, we used a Poisson model with a maximum spatial cluster size of 5.0 percent of the population at risk, meaning the scanning windows can cover up to 5.0 percent of the total expected births/deliveries over the whole study area. We set this value at 5 percent of the population at risk instead of the default 10.0 or 50.0 percent to increase sensitivity to small, localized clusters, as we expected the spatial distribution of facility-based deliveries to operate on a local scale. Larger thresholds (10 or 50 percent) could detect broader patterns but risk masking these finer-scale variations. We conducted two robustness checks with these tow larger thresholds. Overall, the analysis enabled us to pinpoint and investigate the occurrence of high- and low-volume clusters over time and determine whether specific primary healthcare facilities consistently remained clustered over time.

### Geographical analysis of the distribution of conflict deaths in relation to facility-based deliveries over time

To address objective 2, we first subsetted the georeferenced conflict events recorded in the UCDP GED dataset to all events that occurred within Burkina Faso from 2016 to 2021, totalling 673 events resulting in 4,146 conflict-related fatalities. Subsequently, we mapped the spatial distribution of conflict deaths related to these events using a two-dimensional kernel density estimation of the cumulative number of geo-referenced conflict deaths from 2016 to 2017, 2018, 2019, 2020, and 2021, respectively. [39] On top of these cumulative conflict event kernel density layers, we mapped the clusters per year, identified through the spatial scan in the previous stage. The resulting maps enabled us to analyse changes in the clustering of facility-based deliveries over time in relation to the cumulative number of nearby conflict deaths from 2016 to 2021.

### Heterogeneity analysis of the evolution of the rate of facility-based deliveries by pre-conflict service volume and facility type depending on the level of conflict exposure

To address objective 3, we first conducted a service volume analysis in 2018 (i.e., before the main escalation of conflict). To accommodate the varying service volumes of primary healthcare facilities, we conducted a principal component analysis using four output rate indicators (facility-based deliveries + three additional indicators):

- The rate of facility-based deliveries
- The rate of ANC4
- The rate of PNC1
- The rate of curative care visits for children below the age of 15

These indicators are commonly employed in this type of analysis and effectively address maternal and child health concerns [13]. In the subsequent step, to define different service volume levels, we performed a hierarchical ascending classification based on a distance matrix computed using Ward’s method on the results of the principal component analysis. This approach enabled the identification of three categories of primary healthcare centres based on their service volume in 2018 (i.e., before the main escalation of conflict)—namely low, high, and exceptional service volume. Similar to Bonnet and colleagues [13], the aim was to conduct an analysis contextualised within the environment of each facility, thus defining service volume relative to its surroundings, thereby circumventing the necessity to establish fixed thresholds [40]. Identifying the different levels allowed us to analyse whether the rate of facility-based deliveries changed differently over time depending on the pre-conflict service volume levels (low, high, exceptional). Second, we investigated how the average rate of facility-based deliveries changed over time depending on the facility type (CM or CSPS). Finally, to gauge the evolution of disparities in the rate of facility-based deliveries, we computed the interquartile range (IQR) for each month throughout the period. The IQR served as a robust measure of dispersion, particularly adept at handling extreme values. This analysis allowed us to examine whether discrepancies between low-delivery and high-delivery volume facilities widened over time.

We used R software for statistics and GIS, QGIS for mapping, and SatScan for the cluster analysis.

No ethics approval was required by the Ethics Committee of the Medical Faculty of the University of Heidelberg since the study made use exclusively of anonymised secondary data available in open-access modality. Ethical clearance was obtained by Burkina Faso’s Health Research Ethics Committee (CERS), and access to the National Health Information System data was authorised by the Ministry of Health of Burkina Faso (Notice #2021–10–233).

## Results

### Health district analysis

Figure 1 panel A illustrates that, from 2016 to 2021 and within the thick borders, the share of deliveries in CSPSs and CMs sharply declined in the northern health districts. For instance, Titao, Djibo, and Barsalogho experienced reductions ranging from 30 to 50 percent (depicted using a gradient of red tones). In contrast, neighbouring health districts to the south experienced increases ranging from 0 to 20 percent (depicted using a gradient of green tones). Figure 1 panel B reveals that the health district showing declines in panel A also experienced a high number of UCDP conflict deaths during the same period. For instance, Titao, Djibo, and Barsalogho experienced more than 100 conflict deaths between 2016 and 2021. Supplementary material 1 Fig. 3 presents the results of the monthly time series regression at the health district level from January 2013 to December 2021. We found that any conflict-related death was associated with a significant decrease in the rate of facility-based deliveries by 14.2 pp (95% CI: –21.8 to –6.7). Moreover, the negative effects were more severe when the health district experienced a high number of conflict related UCDP deaths, both for more than median exposure and for quintile increases. Based on the results of the health district analysis, we zoomed into the north and east of the country for the remaining analyses of this paper (thick borders in Fig. 1).

### Geographical analysis of the distribution of facility-based deliveries over time

#### Global autocorrelation analysis

Regardless of the method used to identify neighbouring facilities (k = 10 or d = 25), we observed a positive Moran index over the entire study period, which, for k = 10, fluctuated between 0.089 to 0.201 from 2016 to 2021. This result means that the study area is heterogeneous in terms of facility-based deliveries and that there are clusters of primary healthcare centres, that is, groups of primary healthcare centres that are geographically close and share a similar pattern of low or high facility-based deliveries.

#### Spatial scan analysis

**Fig. 2:**
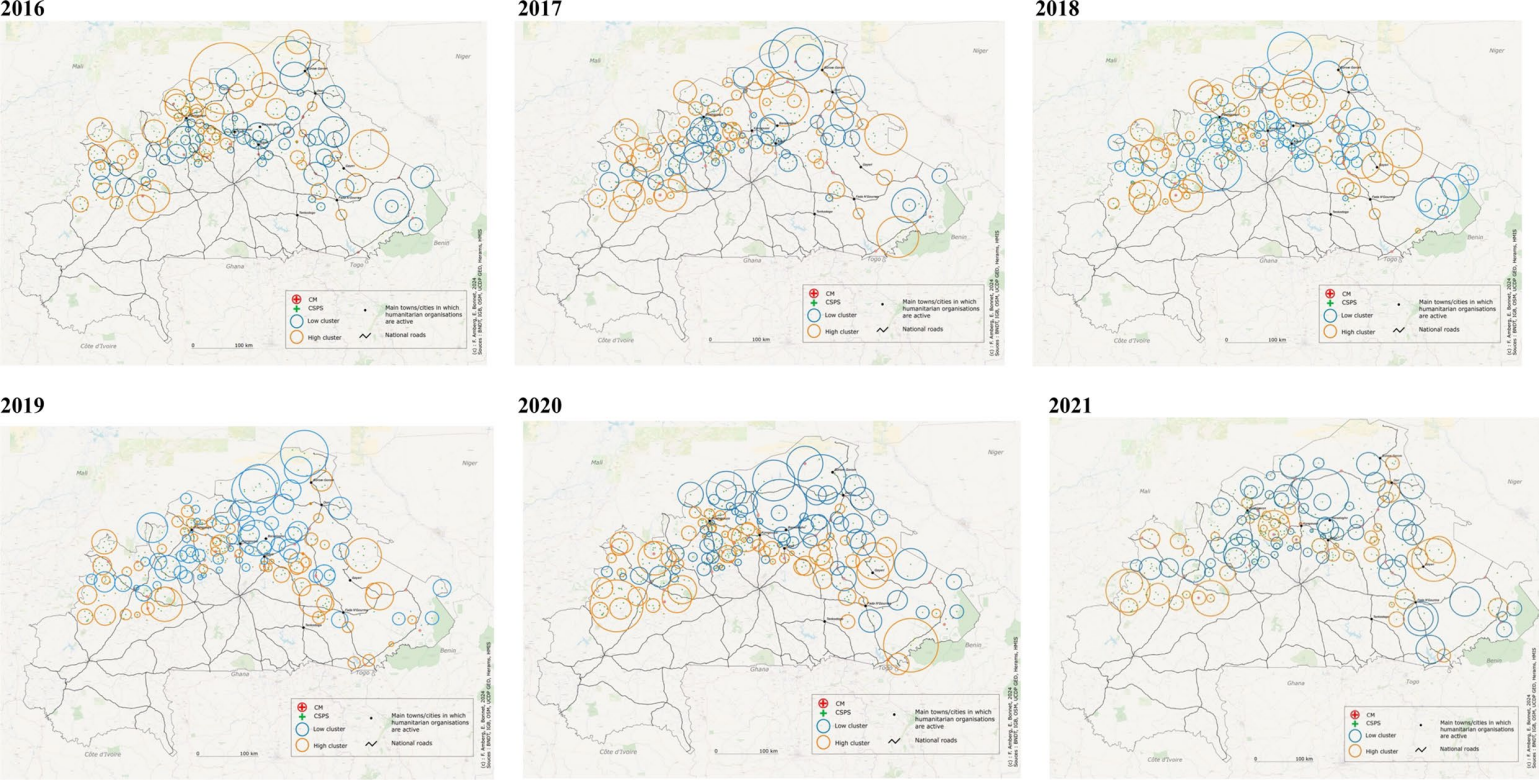
**Spatial scan statistics of the rate of facility-based deliveries. **Statistically significant clusters of facility-based deliveries in CSPS and CM facilities in the zoomed-in area by year, identified using spatial scan statistics with a Poisson model and a maximum cluster size of 5% of the population at risk (see details in methods section). Clustering patterns shifted over time: from 2016 to 2018, a few clusters with higher rates than expected (depicted in orange) were observed in the north, while predominantly clusters with lower rates than expected (marked in blue) were detected further to the south, in the city Kaya and its surroundings. From 2019 onward, low clusters became more common in the north, and high clusters emerged along southern routes and in cities such as Ouahigouya, Kongoussi, Kaya, Gorom-Gorom, Dori, Koupéla, Fada N’Gourma, Gayeri, and Tenkodogo. For map details, refer to Supplementary material 2, where the maps are presented in a larger format

The spatial scan shows that it is not always the same facilities that remain clustered over time. Figure 2 shows that, over the study period, major shifts took place in the occurrence of low and high clusters (groups of facilities that are geographically close and have a lower or higher rate than expected, i.e., if the rates were randomly or uniformly distributed over the study area). Between 2016 and 2018, a few clusters with higher rates than expected (depicted in orange) were observed in the north. Conversely, further to the south, in the city Kaya and its surroundings, predominantly clusters with lower rates than expected (marked in blue) were detected during the same period. However, from 2018 to 2019, a significant change in the occurrence of low and high clusters is evident. From 2019 onwards, low clusters became predominant in the northern areas, while high clusters emerged along the main southern routes and within cities such as Ouahigouya, Kongoussi, Kaya, Gorom-Gorom, Dori, Koupéla, Fada N’Gourma, Gayeri, and Tenkodogo, as well as along connecting roads. In this specific area, the number of high clusters doubled. Initially, between 2016 and 2018, approximately 15 clusters with higher rates than expected were identified. However, this figure increased to 29 by 2019 and 38 by 2021, respectively. The yearly average relative risk also increased between these two time periods. The yearly average relative risk also rose during these periods, from 1.4 between 2016 and 2018 to 1.6 from 2019 to 2021. This implies that between 2019 and 2021, the probability of having more deliveries in these facilities is on average 1.6 times higher than expected. In contrast, in the northern region, the number of low clusters increased over time, from 7 between 2016 and 2018 to approximately 20 between 2019 and 2021. Furthermore, the yearly average relative risk of low clusters in the north decreased from 0.6 (average from 2016 to 2018) to 0.4 (average from 2019 to 2021). In other words, from 2019 to 2021, the observed number of deliveries in northern facilities is 0.4 times the expected number of deliveries.

### Geographical analysis of the distribution of conflict deaths in relation to facility-based deliveries over time

**Fig. 3:**
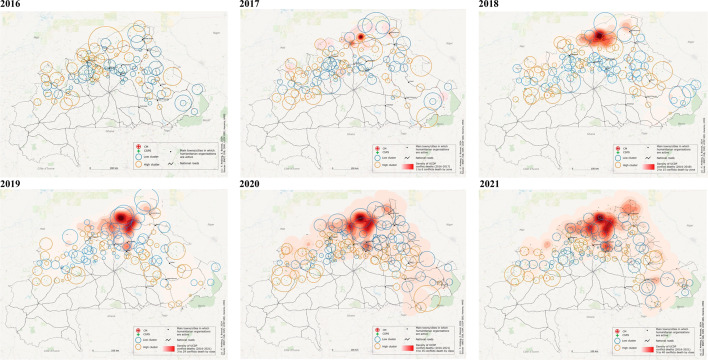
**Geographical analysis of conflict deaths + spatial scan statistics of the rate of facility-based**. Kernel density maps of cumulative UCDP conflict deaths by year overlaid on statistically significant clusters of facility-based deliveries in CSPS and CM facilities in the zoomed-in area by year (identified using spatial scan statistics with a Poisson model and a maximum cluster size of 5% of the population at risk, see details in methods section and Fig. 2). Conflict data are from the UCDP GED dataset. For 2016, there were too few conflict deaths to calculate kernel density. Conflict deaths were unevenly distributed over the territory considered and conflicts intensified mainly in the north of the country and gradually spread further to the south and east, with an escalation of conflict between 2018 and 2019 (Supplementary material 1 Fig. 4 and 5). This escalation of conflict in the north coincided with the noticeable change in the occurrence of clusters of higher and lower rates than expected identified in Fig. 2. From 2019 onwards, clusters with higher rates than expected (depicted in orange) started to appear south of the conflict hotspots along the main southern routes and cities, while within the main conflict zones, we exclusively detected clusters with lower rates than expected (shown in blue) (Supplementary material 1 Fig. 4 and 5). For map details, refer to Supplementary material 2, where the maps are presented in a larger format

Between 2016 and 2021, there were 673 conflict events, resulting in 4,146 conflict-related deaths. However, Fig. 3 shows that the conflict deaths were unevenly distributed over the territory considered and that conflicts intensified mainly in the north of the country and gradually spread further to the south and east. Furthermore, the number of conflict deaths increased sharply between 2018 and 2019 (see also Supplementary material 1 Fig. 4 and 5). This escalation of conflict in the north coincided with the noticeable change in the occurrence of clusters of higher and lower rates than expected identified in the previous analysis. From 2019 onwards, clusters with higher rates than expected (depicted in orange) started to appear south of the conflict hotspots along the main southern routes and cities, while within the main conflict zones, we exclusively detected clusters with lower rates than expected (shown in blue).

### Heterogeneity analysis of the evolution of the rate of facility-based deliveries by pre-conflict service volume and facility type depending on the level of conflict exposure

**Fig. 4:**
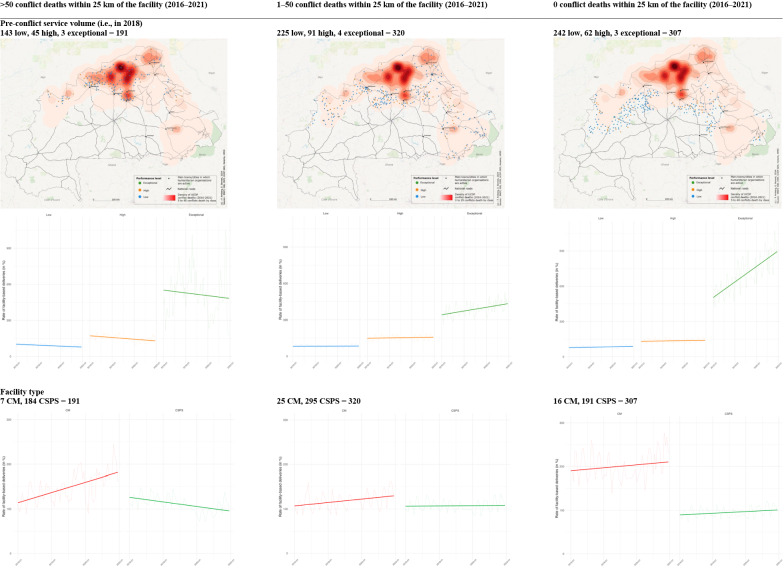
**Change in the monthly rate of facility-based deliveries over time; by conflict exposure, level of pre-conflict service volume (in 2018), and facility type.** The first row depicts the locations of primary healthcare centres by pre-conflict service volume levels (low = blue, high = orange, and exceptional = green) in 2018 (i.e., before the main escalation of conflict) by different levels of conflict exposure within 25 km of the facility between 2016 and 2021 (> 50, 1–50, and 0 conflict deaths). Among facilities with > 50 conflict deaths within 25 km between 2016 and 2021, we identified 143 low (blue), 45 high (orange), and 3 exceptional (green) facilities (total = 191). Among facilities with 1–50 conflict deaths, we detected 225 low, 91 high, and 4 exceptional facilities (total = 320), while there were 242 low, 62 high, and 3 exceptional (total = 307) facilities without any conflict deaths within 25 km from 2016 to 2021. The second row shows the average rate of facility-based deliveries over time for the three different pre-conflict service volume levels in 2018 (low = blue, high = orange, and exceptional = green) and, again, by the different levels of conflict exposure within 25 km of the facility between 2016 and 2021 (> 50, 1–50, and 0 conflict deaths). The three service volume levels were identified through a principal component analysis using four output rate indicators (see details in the methods section). We found declining trends over time for facilities with > 50 conflict deaths within 25 km from 2016 to 2021. For facilities with 0 and 1–50 conflict deaths, we detected increasing trends, which were rather moderate for the low and high, but noticeable for the exceptional 2018 service volume level. The third row plots the average rate of facility-based deliveries over time by facility type (CM = red, CSPS = green, colour as in facility type symbols in Fig. 2 and 3) and, again, by the different levels of conflict exposure within 25 km of the facility between 2016 and 2021 (> 50, 1–50, and 0 conflict deaths). Facilities situated in conflict hotspots (i.e., > 50 conflict deaths within a 25 km between 2016 and 2021) showed contrasting trajectories. Specifically, CM facilities displayed an upward trend, while CSPS experienced a decline. For lower nearby conflict exposure (< 50 or 0 conflict deaths), both facility types showed an upward trend, which is, however, in both cases, more noticeable for CM facilities. For map details, refer to Supplementary material 2, where the maps are presented in a larger format

In Fig. 4 panel A, we calculated and mapped the pre-conflict service volume level of each facility, separately for different levels of conflict exposure (0, 1–50, > 50 conflict deaths reported in a geographical area within a 25 km radius of the facility between 2016 and 2021) and on top of the kernel density estimation from the previous step. In 2018, among facilities with more than 50 conflict deaths within 25 km between 2016 and 2021, we identified 143 low (blue), 45 high (orange), and 3 exceptional (green) facilities (total = 191). Among facilities with 1–50 conflict deaths between 2016 and 2021, we detected 225 low, 91 high, and 4 exceptional facilities (total = 320), while there were 242 low, 62 high, and 3 exceptional (total = 307) facilities without any conflict deaths within 25 km from 2016 to 2021. When examining how the average rate of facility-based deliveries changed over time depending on the level of pre-conflict service volume (low, high, or exceptional) and conflict exposure (0, 1–50, or > 50 conflict deaths within 25 km of the facility between 2016 and 2021), we found declining trends over time for facilities with more than 50 conflict deaths within 25 km from 2016 to 2021. For facilities with 0 and 1–50 conflict deaths, we detected increasing trends, which were rather moderate for the low and high, but noticeable for the exceptional 2018 service volume level.

When analysing the rate by facility type and conflict exposure (Fig. 4 panel B), our analysis revealed that facilities situated in conflict hotspots (i.e., > 50 conflict deaths within a 25 km between 2016 and 2021) showed contrasting trajectories. Specifically, CM facilities displayed an upward trend, while CSPS experienced a decline. For lower nearby conflict exposure (< 50 or 0 conflict deaths), both facility types showed an upward trend, which is, however, in both cases, more noticeable for CM facilities.

Finally, as indicated by the change in the IQR of the rate of facility-based deliveries (Supplementary material 1 Fig. 6), the overall inequality in the rate of facility-based deliveries tended to increase during the period under consideration. The increase in this gap indicates a divergence between the 25% of facilities with the lowest rates and the 25% with the highest rates. In short, the gap is gradually widening between the facilities with the lowest and highest delivery volume.

## Discussion

In Burkina Faso, the escalation of conflict has had profound implications for healthcare access, particularly obstetric care [9, 10]. This is the first study to provide crucial insights into the spatial evolution of facility-based deliveries at primary healthcare centres amidst the security crisis and at the finest possible scales over time. We examined how facility-based delivery rates evolved spatially over time and across different locations, in relation to the proximity and intensity of conflict. Additionally, we examined how facility characteristics and spatial determinants combined to influence the patterns we observed. Thereby, our research offers insights into the potential to strengthen Burkina Faso’s health system resilience to conflict. By mapping healthcare disruptions due to nearby conflict and identifying facility characteristics and geo-spatial factors shaping resilience, our work generates evidence for adaptive policy intervention and dynamic resource allocation based on conflict dynamics, thereby enhancing health facilities’ ability to sustain obstetric care during conflict. Furthermore, with a few exceptions [6, 7, 10, 16], most existing studies concerning access to maternal healthcare in conflict settings typically take a broad view, examining data at the regional, district, or commune scale [9, 41–45]. Yet, by scrutinizing a finer geographical context, this study exposes the heterogeneous characteristics of conflict-affected regions. Our analysis revealed that as the conflict escalated, facility-based delivery rates concentrated in facilities located along major southern routes and cities more frequently than in northern regions. Furthermore, contrasting trends were noted between CSPS and CM facilities within conflict zones, with CM facilities showing an upward trajectory in the monthly rate of facility-based delivery, indicating that they were more resilient to the effects of conflict. Outside conflict areas, CM facilities also maintained higher facility-based delivery rates over time. Hereafter, we discuss in some detail the main findings, starting with a discussion of the effect of conflict in hotspot areas, followed by an appraisal of what may explain the spatial shift we observe over time, and we conclude by gauging the factors shaping health facility resilience in times of conflict and insecurity in Burkina Faso.

Our findings showed that the impact of conflict on facility-based deliveries is particularly pronounced in conflict hotspots in the north, areas characterized by a persistent decline in delivery rates over time, irrespective of the pre-conflict service volume. In general, the lower rates of facility-based deliveries observed in conflict hotspots compared to other locations can be attributed to the fact that delivery care demands considerable resources and relies heavily on infrastructures, connectivity to the national transportation networks, medical supply chains, and the presence of trained medical personnel [10]. Hence, disruptions in the healthcare system caused by nearby conflict events, such as fuel and electricity shortages, material scarcities, staff absenteeism, and the unavailability of medical transportation to district hospitals can lead to a collapsing health system within conflict hotspots. The insecurity and direct attacks on rural healthcare facilities have forced health workers to leave the affected areas or reduce their activities, leaving many healthcare facilities unable to function [11–13]. Furthermore, in areas under attack, terrorist groups frequently withdraw ambulances, hindering the evacuation of women [29]. In addition, deliveries and obstetric emergencies can occur at night when insecurity typically peaks and when even the facilities that manage to open during daytime mostly stop operating due to higher insecurity [46]. In the case where the nearby healthcare facility still operates, this situation can also prompt women to choose delivering in their villages.

The spatial shift in where facility-based deliveries occur over time underscores geographical variation in facility-based deliveries across the study area. The emergence of high clusters in relatively safer southern areas can reflect both a redirection of demand and supply of delivery care towards urban centres, a finding which is consistent with prior evidence from Mali [13]. Due to the nature of the data used in our study, it was impossible to differentiate the extent to which the shifts we observed were predominantly due to a redirection of demand or supply for delivery care. Yet, existing literature offers evidence for both sides of this complex interplay. Insecurity has led to population displacement throughout the Sahel region, with affected individuals seeking refuge in camps with limited healthcare resources or fleeing to (semi-)urban centres [47]. For instance, a significant portion of the non-nomadic population—particularly the Mossi and Yarcé communities—who had been settled in the north for decades, have fled southward to areas such as Kaya, Kongoussi, Titao, and Ouahigouya, where the government still maintains control. This displacement resulted in a redirection of demand for delivery services among pregnant IDPs, who often seek safer, more accessible health facilities away from conflict zones. On the supply side, our finding resonates with Ouedraogo and colleagues who demonstrated that due to the closure and limited operation of healthcare facilities in regions facing security challenges in the north, there is a concentration of healthcare personnel in urban centres towards the south, particularly in the regional and district capitals [48]. Due to the progressive loss of state authority, the north has been increasingly cut off, leading to a breakdown and isolation of essential health services in these areas.

It should be highlighted that what emerges most clearly from the analysis is not evidence of robust system-wide resilience but rather a redistribution of facility-based deliveries across areas with differing security levels, reflecting predictable patterns of population displacement away from zones of acute threat. Furthermore, the design of this study does not allow to draw any conclusions about the quality of care provided, which in such circumstances often deteriorates. Finally, evidence from a comparable context in neighbouring Mali shows only *minimal resilience*. [49] In that setting, coping mechanisms remained basic, fragmented, and heavily dependent on personal commitment rather than institutional support, as primary health centres lacked coordinated or system-level strategies to withstand conflict pressures, with resilience emerging only from the response of individuals—often poorly trained and working under difficult conditions with high workload and minimal operational flexibility. In the most isolated areas, working conditions deteriorated to such an extent that fear and fatigue led to staff attrition. Consequently, any attribution of resilience to Burkina Faso’s system should be treated with caution. Given the similarities in health system structures and conflict dynamics, it is plausible that the resilience observed in our study is also only minimal, with service continuity maintained in some facilities, but at the expense of quality and through unsustainable individual-level absorption. Nevertheless, certain facility-level traits helped sustain service delivery and prevent systemic collapse. Hereafter, we outline these factors.

First, facilities located outside the main conflict hotspots, particularly those near major southern routes and larger urban areas tend to demonstrate greater resilience. Despite receiving a lot of internally displaced people and having only limited additional human resources, they made a significant contribution to maintaining delivery care. These locations likely benefit from better security, infrastructure, and accessibility, making them less vulnerable to disruptions caused by armed conflict. Second, facilities with high or exceptional pre-conflict service volumes outside conflict zones were notably more resilient, suggesting that larger, well-established facilities with a higher patient throughput are better positioned to adapt to operational challenges. This resilience may be due to more robust infrastructure and better resource allocation, allowing them to cope with the demands of conflict more effectively than smaller, less resourced facilities. Finally, CM facilities, both within and outside conflict-affected areas, exhibit a higher capacity to maintain delivery services over time compared to CSPS facilities. This disparity can be attributed to several factors. First and most importantly, CMs are usually in (semi-) urban areas, whereas CSPSs are mostly located in rural areas, and insecurity in Burkina Faso tends to be higher in proximity to rural facilities in the north as opposed to (semi-)urban facilities towards the south [29]. CMs demonstrate greater capacity to maintain service delivery and manage to cope with the increasing burden imposed on them by the closure or malfunctioning of conflict-affected CSPSs. Although medium-sized, semi-urban towns towards the south where CMs are typically located also experience attacks, terrorist groups often lack the power to fully occupy these areas as they often do with villages in the north. In some (semi-)urban areas to the south, military detachments or self-defense groups are present, offering a certain level of security. As a result, health workers remain in place, continuing to provide care for displaced persons [47]. Second, in (semi-)urban areas in the north where terrorist groups actually managed to seize control, terrorist groups have a tactical interest in ensuring that larger urban health facilities remain operational—despite multiple attacks carried out while seizing control of these areas in Burkina Faso [11, 12]. This pattern was also observed by health personnel in a comparable conflict setting in neighbouring Mali [13]. For instance, the groups often have local informal agreements or deals with the populations they control to maintain at least some level of care through larger facilities. Moreover, when terrorist groups expand into (semi-)urban areas, they are typically slow to target health workers, whose services they may depend on. In some cases, they have even kidnapped health personnel and brought them to their camps to care for injured combatants [50]. This underscores the critical role of CMs in maintaining access to healthcare, regardless of who controls the area. Third, the presence of a doctor can also explain sustained higher rates of facility-based deliveries in conflict-affected CMs compared to conflict-affected CSPSs. In volatile situations, people may be more inclined to use facilities with doctors (MDs) just in case something goes wrong during delivery. In the awareness that the local CSPS would not be able to handle the case, or might even be closed when the time comes, and that transportation to a higher-level facility in case of complications might be impossible, women might be more inclined than in peaceful times to be near a CM or even higher-level facility when the due date approaches. Another reason, and perhaps more significant than the mere presence of a doctor, could be sheer size, as CMs are larger and often handle a higher patient volume than CSPSs. They typically have more personnel, both in terms of quantity and qualifications, and more infrastructure, making them better prepared for a conflict shock and better able to resist. In contrast, in conflict-affected CSPS facilities, the limited staff (usually only 2 or 3 nurses or midwives) are more likely to leave under difficult conditions, whereas CMs benefit from a greater likelihood of maintaining health professionals on site due to their larger workforce. Lastly, there may be informal decisions to allocate more resources to CMs compared to CSPSs, possibly more so during conflict periods as CMs naturally have also more funds than CSPSs, given the higher service volume they offer.

## Limitations

Our study, while providing valuable insights into the challenges of accessing healthcare in conflict-affected regions, is subject to several limitations. First, the nature of the data used limits our ability to differentiate between disruptions caused by demand-side factors, such as decreased healthcare-seeking behaviour, and supply-side factors, such as reduced availability of healthcare services. Second, there is a possibility of selection bias due to missing data, particularly in conflict-affected areas where data reporting is challenging and not prioritised, potentially leading to underestimation of the true impact of conflict on healthcare access. Third, as explained above, the methods used in this study did not allow for the inclusion of higher-tier referral hospitals within the health system hierarchy, such as Centres Médicaux avec Antenne Chirurgicale (CMAs) and Centres Hospitaliers Régionaux (CHRs). If such an inclusion had been possible, it could have further enhanced the understanding of how conflict affects obstetric care. Fourth, the lack of data on humanitarian NGO activities and the incomplete reporting of private healthcare providers in routine health management information system (HMIS) data—leading to their exclusion from this study—may result in an underestimation of overall healthcare coverage in conflict zones. Fifth, our study lacks data on internal displacement at the geographical resolution required for precise geospatial analysis, leading to potential inaccuracies in population estimates and rates of facility-based deliveries. Sixth, the geographical methods used in this paper rely on assumptions and are sensitive to parameter choices. However, in sensitivity analyses with different parameter choices (e.g., d = 10 and k = 5 as well as d = 50 and k = 20 vs. d = 25 and k = 10 in the global autocorrelation analysis, Poisson model with a maximum spatial cluster size of 50.0 and 10.0 percent vs. 5.0 percent in the spatial scan analysis), we obtained very similar results (positive Moran index over the entire study period, same spatial shift in where facility-based deliveries occur over time). Finally, while we discussed geo-spatial factors, we did not conduct a detailed analysis of the physical environment. Key factors such as urban dispersion, geography, the transportation infrastructure, and the presence and dynamics of IDP camps and NGO support are not directly modelled in our analysis. Future research should incorporate these dimensions to provide a more comprehensive understanding of healthcare access in conflict-affected areas.

## Conclusion (implications and so what?)

The study’s findings have significant implications for health policy planning in conflict-affected regions, offering a roadmap for resilient healthcare adaptation. The observed shift in facility-based deliveries toward safer urban areas and major southern routes highlights the urgent need for an adaptive healthcare strategy that dynamically allocates resources based on evolving conflict dynamics. By leveraging geospatial analyses, this study demonstrates how fine-scale conflict and health data can inform such a conflict-sensitive maternal health planning. In practice, these methods can be integrated into a national health management system, enabling near real-time spatial tracking of healthcare disruptions caused by nearby conflict. Such a system would enhance health system responsiveness by facilitating timely interventions, directing funding, deploying personnel, and delivering medical supplies to the most affected areas. Moreover, geospatial mapping can improve the accuracy of healthcare demand forecasting, ensuring a more proactive and data-driven approach to crisis response.

Although, in conflict settings, the implementation of measures to enhance facility-based and assisted deliveries is challenging, the findings of this study provide robust evidence for policy and health system planning. Numerous obstacles, as outlined in prior literature reviews focusing on low- and middle-income countries (LMICs) [51, 52], include security concerns, insufficiently trained healthcare personnel, and financial constraints [53, 54]. Nonetheless, despite these hurdles, researchers and local health systems have developed innovative delivery strategies and policies that improve health system resilience in times of conflict. [55, 56] A number of interventions developed through these initiatives may hold relevance for addressing the spatial shifts in facility-based delivery rates. First, policymakers should focus on strengthening healthcare infrastructure, particularly in conflict-prone zones. Our findings highlight the need for a dual investment strategy. Supporting CSPSs is crucial to prevent their collapse, ensuring that healthcare services remain accessible to rural populations who are unable to relocate. Likewise, it is important to support CMs to handle the increased burden some of them may still face if nearby conflict-affected CSPSs cannot be sufficiently supported to prevent closure. This includes investments in staffing, supply chains, and security measures. At the same time, resilience may depend not only on investments in infrastructure, which remain highly vulnerable in conflict settings, but also on incentive-based approaches that encourage health workers to remain in place or to relocate to facilities under strain. Second, tailored strategies, such as the delivery of integrated service packages at the point of care and community outreach programs, are essential to meet the unique needs of populations in the conflict hotspots. Albeit with the risk of not sufficiently ensuring the quality of services provided, the efforts of the Ministry of Health, such as reactivating village birth attendants, establishing advanced health posts and deploying community health workers, contribute to maintaining continuity of care and demonstrate some of the targeted measures already being implemented [29, 30]. Finally, the initiatives point out that collaboration between humanitarian agencies, government authorities, and local communities could support these efforts.

While our quantitative analysis sheds light on the patterns of healthcare access, supportive qualitative analyses are needed to differentiate the extent to which disruptions are due to demand or supply factors and to provide deeper understanding of the resilience factors. These qualitative analyses could involve key informant interviews and focus group discussions with stakeholders across various sectors and administrative levels (national, regional, district, and facility level), and interviews with affected populations, provided the security situation permits such data collection efforts.

Moving forward, policymakers and healthcare stakeholders in Burkina Faso can utilise these insights to develop targeted, resilient, flexible, and context-specific interventions or improve strategies that have already been implemented for equitable healthcare access in the challenging environment.

## Supplementary Information

Supplementary material file 1

Supplementary material file 2

## Data Availability

Data are available in a public, open access repository. Data may be obtained from a third party and are not publicly available. Data from the Uppsala Conflict Data Program Georeferenced Event Dataset (UCDP GED) are publicly available online (https://ucdp.uu.se/). Data from the National Health Information System can be obtained by contacting the Ministry of Health of Burkina Faso.

## Declarations

### Patient and public involvement statement

Patients and members of the public were not involved in the design, conduct, reporting and dissemination of this research. Utilisation of healthcare services by patients were routinely collected by healthcare facility providers, fed into the National Health Management Information System, and only as such, analysed by the study team; data were aggregated, and individual patients cannot be identified from the reported data.

### AI use statement

The AI technology ChatGPT was used to improve word processing and language.
