# Correction: The evolution of facility-based deliveries at primary healthcare centres during an insecurity and conflict crisis in Burkina Faso: a geospatial analysis

**DOI:** 10.1186/s13031-025-00731-8

**Published:** 2025-12-05

**Authors:** Felix Amberg, Manuela De Allegri, Valéry Ridde, Ali Sie, Mariam Seynou, Kadidiatou Kadio, Sayouba Dianda, Julia Lohmann, Karl Blanchet, Neha S. Singh, Emmanuel Bonnet

**Affiliations:** 1 Heidelberg Institute of Global Health, Heidelberg University Hospital and Medical Faculty, Heidelberg University, Heidelberg, Germany; 2 Institut de Recherche pour le Développement, Centre Population et Développement, Université Paris Cité, Paris, France; 3 Centre de Recherche en Santé de Nouna, Nouna, Burkina Faso; 4 Institut de Recherche en Science de la Santé, Ouagadougou, Burkina Faso; 5 University of Bonn, Bonn, Germany; 6 Institute for Medical Information Processing, Biometry, and Epidemiology, Ludwig Maximilian University of Munich, Munich, Germany; 7 Geneva Centre of Humanitarian Studies, Faculty of Medicine, University of Geneva, Geneva, Switzerland; 8 Department of Global Health and Development, Faculty of Public Health and Policy, London School of Hygiene & Tropical Medicine, London, UK; 9 Institut de Recherche pour leDéveloppement, Centre National de la Recherche Scientifique, Université Paris, Paris, France

**Correction: Conflict and Health (2025) 19:78**

10.1186/s13031-025-00723-8

In this article Fig. 4 appeared incorrectly and have now been corrected in the original publication. For completeness and transparency, the correct and incorrect versions are displayed below.

Incorrect Fig. 4


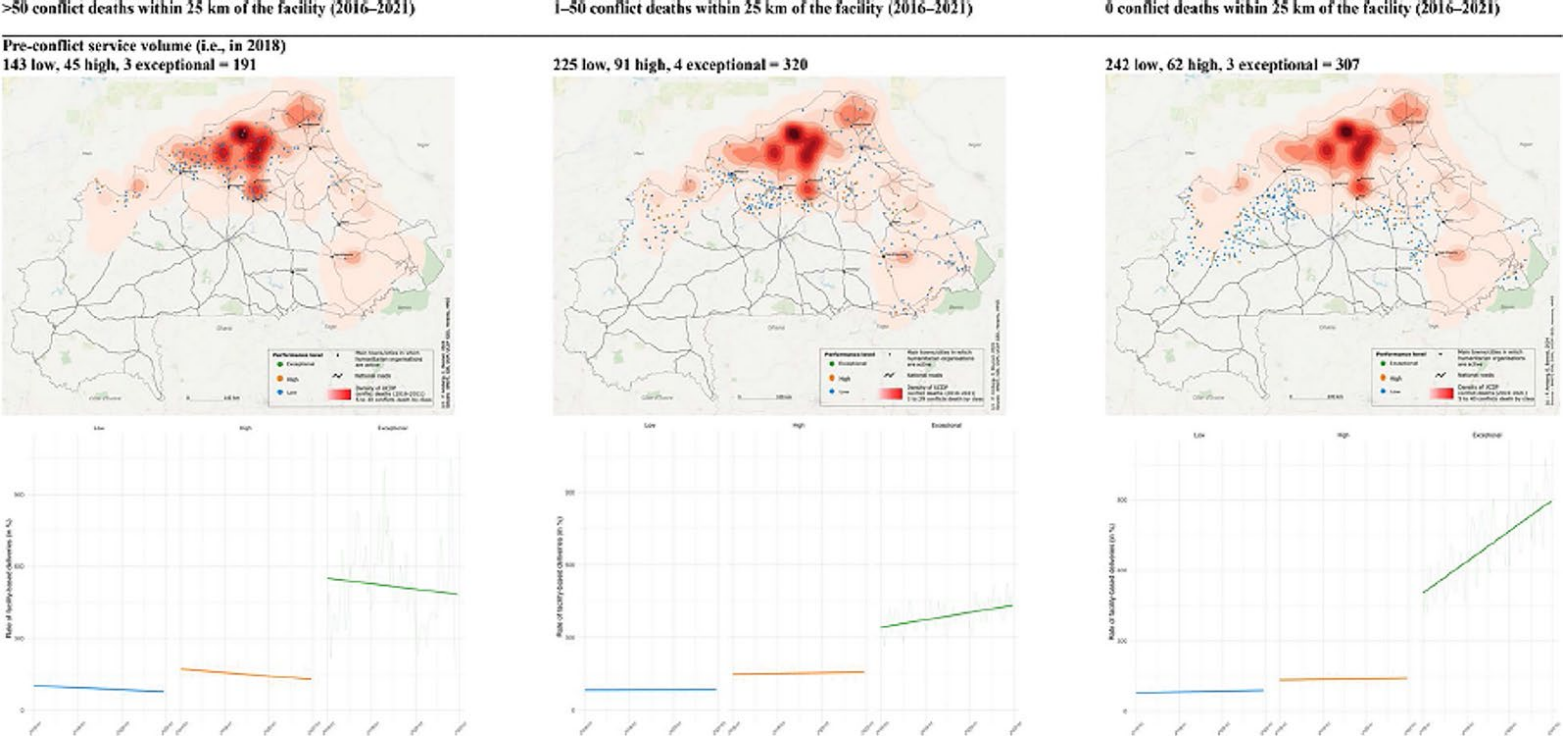


Fig. 4 Change in the monthly rate of facility-based deliveries over time; by conflict exposure, level of pre-conflict service volume (in 2018), and facility type. The first row depicts the locations of primary healthcare centres by pre-conflict service volume levels (low = blue, high = orange, and exceptional = green) in 2018 (i.e., before the main escalation of conflict) by different levels of conflict exposure within 25 km of the facility between 2016 and 2021 (>50, 1–50, and 0 conflict deaths). Among facilities with >50 conflict deaths within 25 km between 2016 and 2021, we identified 143 low (blue), 45 high (orange), and 3 exceptional (green) facilities (total = 191). Among facilities with 1–50 conflict deaths, we detected 225 low, 91 high, and 4 excep tional facilities (total = 320), while there were 242 low, 62 high, and 3 exceptional (total = 307) facilities without any conflict deaths within 25 km from 2016 to 2021. The second row shows the average rate of facility-based deliveries over time for the three different pre-conflict service volume levels in 2018 (low = blue, high = orange, and exceptional = green) and, again, by the different levels of conflict exposure within 25 km of the facility between 2016 and 2021 (>50, 1–50, and 0 conflict deaths). The three service volume levels were identified through a principal component analysis using four output rate indicators (see details in the methods section). We found declining trends over time for facilities with >50 conflict deaths within 25 km from 2016 to 2021. For facilities with 0 and 1–50 conflict deaths, we detected increasing trends, which were rather moderate for the low and high, but noticeable for the exceptional 2018 service volume level. The third row plots the average rate of facility-based deliveries over time by facility type (CM = red, CSPS = green, colour as in facility type symbols in Figs. 2 and 3) and, again, by the different levels of conflict exposure within 25 km of the facility between 2016 and 2021 (>50, 1–50, and 0 conflict deaths). Facilities situated in conflict hotspots (i.e., >50 conflict deaths within a 25 km between 2016 and 2021) showed contrasting trajectories. Specifically, CM facilities displayed an upward trend, while CSPS experienced a decline. For lower nearby conflict exposure (< 50 or 0 conflict deaths), both facility types showed an upward trend, which is, however, in both cases, more noticeable for CM facilities

Correct Fig. 4

**Fig. 4:**
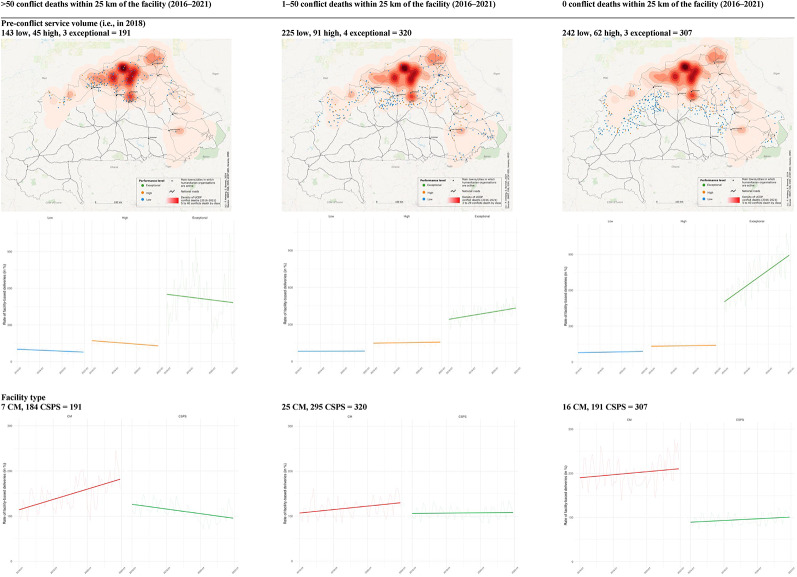
Change in the monthly rate of facility-based deliveries over time; by conflict exposure, level of pre-conflict service volume (in 2018), and facility type. The first row depicts the locations of primary healthcare centres by pre-conflict service volume levels (low = blue, high = orange, and exceptional = green) in 2018 (i.e., before the main escalation of conflict) by different levels of conflict exposure within 25 km of the facility between 2016 and 2021 (>50, 1–50, and 0 conflict deaths). Among facilities with >50 conflict deaths within 25 km between 2016 and 2021, we identified 143 low (blue), 45 high (orange), and 3 exceptional (green) facilities (total = 191). Among facilities with 1–50 conflict deaths, we detected 225 low, 91 high, and 4 excep tional facilities (total = 320), while there were 242 low, 62 high, and 3 exceptional (total = 307) facilities without any conflict deaths within 25 km from 2016 to 2021. The second row shows the average rate of facility-based deliveries over time for the three different pre-conflict service volume levels in 2018 (low = blue, high = orange, and exceptional = green) and, again, by the different levels of conflict exposure within 25 km of the facility between 2016 and 2021 (>50, 1–50, and 0 conflict deaths). The three service volume levels were identified through a principal component analysis using four output rate indicators (see details in the methods section). We found declining trends over time for facilities with >50 conflict deaths within 25 km from 2016 to 2021. For facilities with 0 and 1–50 conflict deaths, we detected increasing trends, which were rather moderate for the low and high, but noticeable for the exceptional 2018 service volume level. The third row plots the average rate of facility-based deliveries over time by facility type (CM = red, CSPS = green, colour as in facility type symbols in Figs. 2 and 3) and, again, by the different levels of conflict exposure within 25 km of the facility between 2016 and 2021 (>50, 1–50, and 0 conflict deaths). Facilities situated in conflict hotspots (i.e., >50 conflict deaths within a 25 km between 2016 and 2021) showed contrasting trajectories. Specifically, CM facilities displayed an upward trend, while CSPS experienced a decline. For lower nearby conflict exposure (< 50 or 0 conflict deaths), both facility types showed an upward trend, which is, however, in both cases, more noticeable for CM facilities

The original article has been corrected.

