# Supplementary material for: The evolution of facility-based deliveries at primary healthcare centres during an insecurity and conflict crisis in Burkina Faso: a geospatial analysis
Source: Confl Health. 2025 Nov 3;19:78. doi: 10.1186/s13031-025-00723-8 (PMC12581450; doi:10.1186/s13031-025-00723-8)
Supplement: Supplementary file 1 — Supplementary material file 1 [file 13031_2025_723_MOESM1_ESM.pdf]

The evolution of facility-based deliveries at primary healthcare centres during an insecurity and conflict crisis in Burkina Faso: a geospatial analysis

Felix Amberg, Manuela De Allegri, Valéry Ridde, Ali Sie, Mariam Seynou, Kadidiatou Kadio, Sayouba Dianda, Julia Lohmann, Karl Blanchet, Neha S. Singh, Emmanuel Bonnet

Supplemental Material

April 14, 2025

Contents

1 Critical data cleaning steps **4**

2 Defining the target population living around the CSPSs and CMs **5**

3 Monthly health district level time series analysis throughout Burkina Faso **7**

4 Conflict-related deaths by type of violence over time, 2016–2021 **8**

5 Distribution of CSPS and CM facilities (left) and conflict events (right) in Burkina Faso, 2016–2021 **9**

6 Distribution of CSPS and CM facilities (left) and conflict events (right) in Burkina Faso, 2016–2021 **10**

7 Trend in the interquartile range of facility-based deliveries **11**

List of Figures

S1 Voronoi polygons around the CSPSs and CMs 5
S2 Grid population estimates from WorldPop within each facility's voronoi polygon 6
S3 The effect of conflict deaths on the rate of facility-based deliveries (January 2013–December 2021) . 7
S4 Conflict-related deaths by type of violence over time, 2016–2021 8
S5 Distribution of CSPS and CM facilities (left) and conflict events (right) in Burkina Faso, 2016–2021 . 9
S6 Distribution of CSPS and CM facilities (left) and conflict events (right) in Burkina Faso, 2016–2021 . 10
S7 Trend in the interquartile range of facility-based deliveries 11

1. Critical data cleaning steps

Outlier detection

We have addressed outliers in the monthly service counts across all facilities. To identify these outliers, we employed a rolling modified Z-score method within each facility, considering the preceding and subsequent 24 months in each iteration. An outlier was defined as having a rolling modified z-score exceeding 15. In their 1993 work, Iglewicz and Hoaglin (1) recommend a threshold of 3.5 for the modified Z-score to detect outliers. However, this threshold is not universally applicable; it should be adjusted on the basis of the specific characteristics of the dataset and the objectives of the analysis. In our study, we opted for a more conservative threshold of 15. This decision was informed by empirical evaluations, in which we examined the trajectories of facilities flagged as having an outlier under various thresholds. We observed that the standard threshold of 3.5 identified an excessive number of outliers, often marking nearly every facility trajectory as anomalous, which did not align with realistic expectations. By increasing the threshold to 15, we effectively isolated only the most conspicuous outliers, thereby enhancing the reliability of our outlier detection process. Once identified, outliers were corrected through a rolling median approach within each facility, taking into account the previous and following 12 months in each iteration.

Imputation of missing values

Additionally, we handled internal missing counts for facilities meeting specific criteria: those with a maximum of 10 consecutive missing counts and at least 10 non-missing values throughout the study period. Imputation of missing values within each facility was carried out using a nonparametric estimator called the local polynomial smoother (LPS). In the LPS-based regression, a low degree polynomial is fitted, through weighted least squares, not to the whole time series but to temporal segments localised by a kernel (or moving window) with specific bandwidth (or smoothing parameter). The imputation technique can be effectively used to estimate the regression function for diverse types of time series,(2; 3) which is the case with our data, as the trajectories of the different institutions differ greatly. This step was crucial due to the health sector crisis in Burkina Faso during 2019, which led to a lack of statistical reports from April to November. Consistent with established literature on handling missing data, the approach aimed to minimise bias in our analyses.(4; 5; 6; 7; 8)

2. Defining the target population living around the CSPSs and CMs

1. **Generate Voronoi polygons around the CSPSs and CMs for all facilities in a given year**

   We first generated Voronoi polygons around the CSPSs and CMs for all facilities in a given year (i.e., depending on how many healthcare facilities were active each year). Voronoi polygons are partitions of a space into regions that are determined by a given set of points in space, referred to here as facilities. In other words, by generating Voronoi polygons, we partitioned the study area into areas for which the respective healthcare facilities are responsible.

   Here, we just give an example with all facilities active during the time period from 2016 to 2021. In the paper, we generate Voronoi polygons for each year individually (i.e., depending on how many health facilities were active each year).

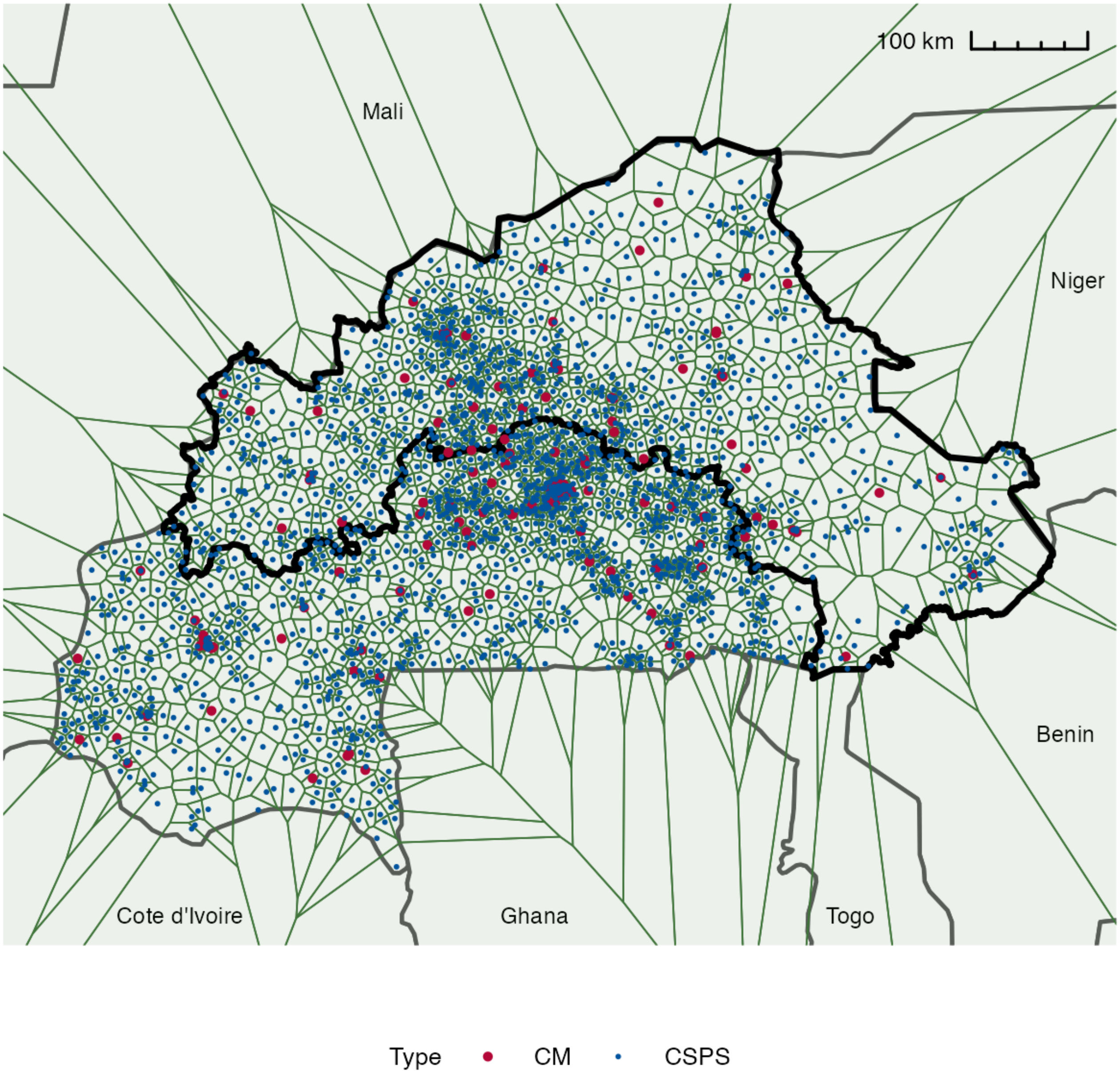

Figure S1: Voronoi polygons around the CSPSs and CMs

The area examined in the geospatial analyses is displayed with thick borders.

2. **Sum up grid population estimates from WorldPop within each voronoi polygon**

   Next, we summed up grid population estimates from WorldPop within each voronoi polygon.(9) Subsequently, to get the number of expected births/deliveries, we multiplied the total number of people living in these areas by Burkina Faso's yearly crude birth rate (from the World Bank). We estimated the target population around the facilities to finally determine the rate of facility-based deliveries by dividing the number of facility-based deliveries by the number of expected births/deliveries in the surroundings of the facility.

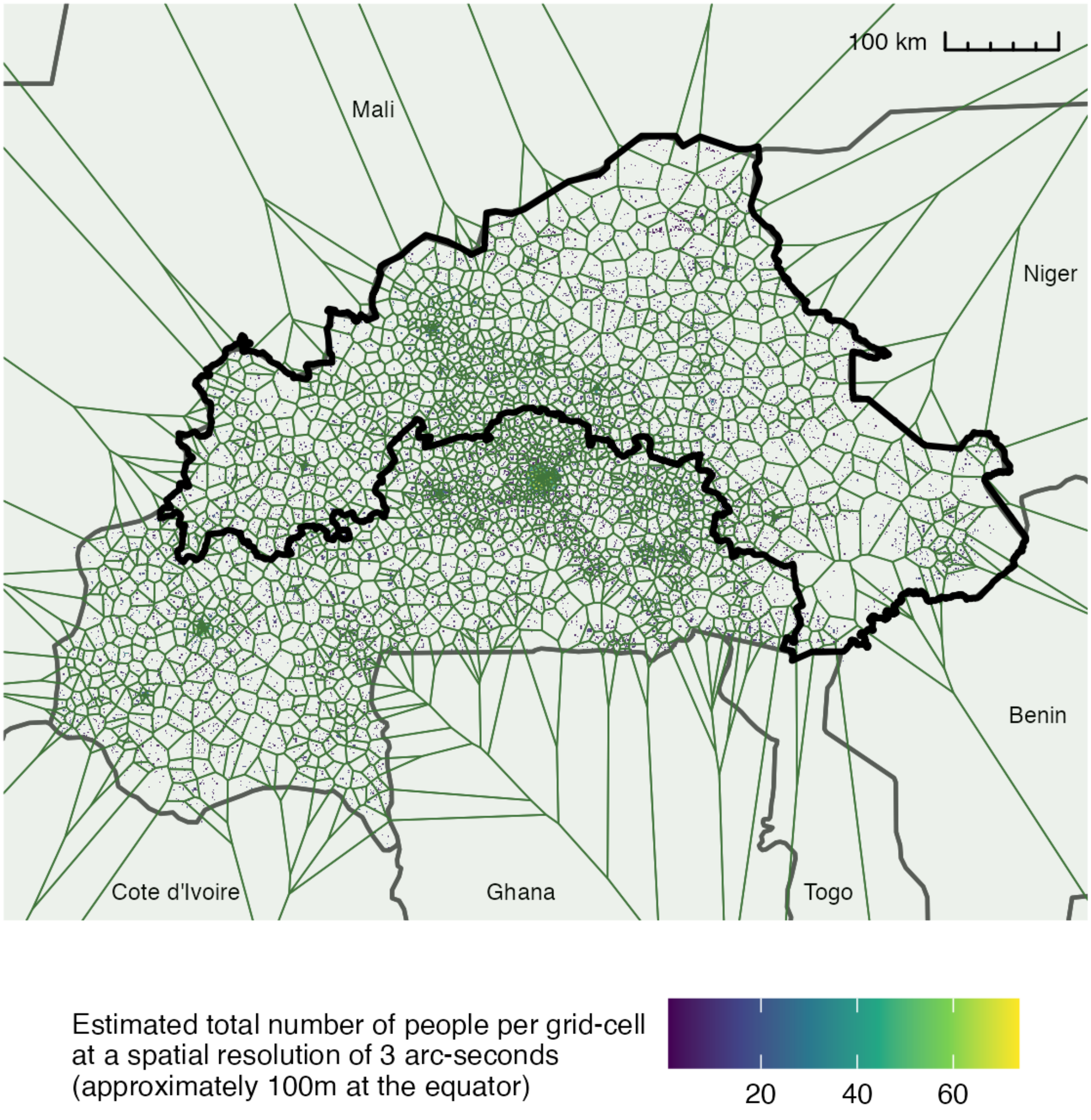

Figure S2: Grid population estimates from WorldPop within each facility's voronoi polygon

The area examined in the geospatial analyses is displayed with thick borders.

3. Monthly health district level time series analysis throughout Burkina Faso

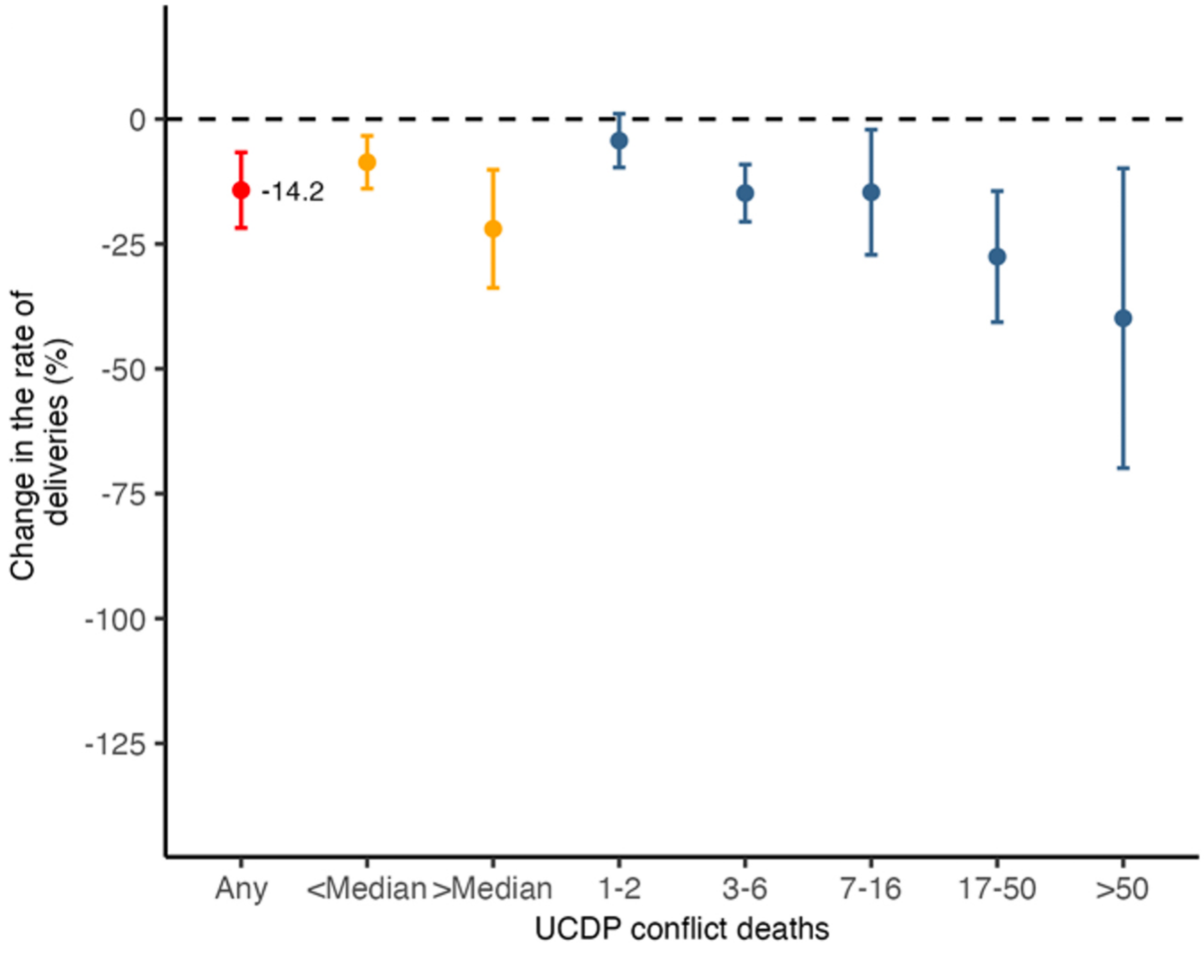

Figure S3: Monthly health district level time series analysis throughout Burkina Faso: the effect of conflict deaths on the rate of facility-based deliveries (January 2013–December 2021)

This figure shows the effect of any conflict deaths (red bar) and conflict exposure of increasing intensity [orange bars represent below- and above-median intensity (¿7 deaths), blue bars depict the quartiles of the number of conflict deaths, as well as one indicator for a very high number of conflict deaths (¿50 deaths)] in the month of health service access at the health district level (n=71). The y axis indicates the decrease in percentage points in the rate of facility-based deliveries. The nominator is obtained from HMIS and, unlike in the geospatial analyses at the facility level, for this analysis, the denominators (i.e., health district population estimates) are sourced from the annual HMIS statistics. Error bars represent 95% CIs, corresponding to p¡0·05.

4. Conflict-related deaths by type of violence over time, 2016–2021

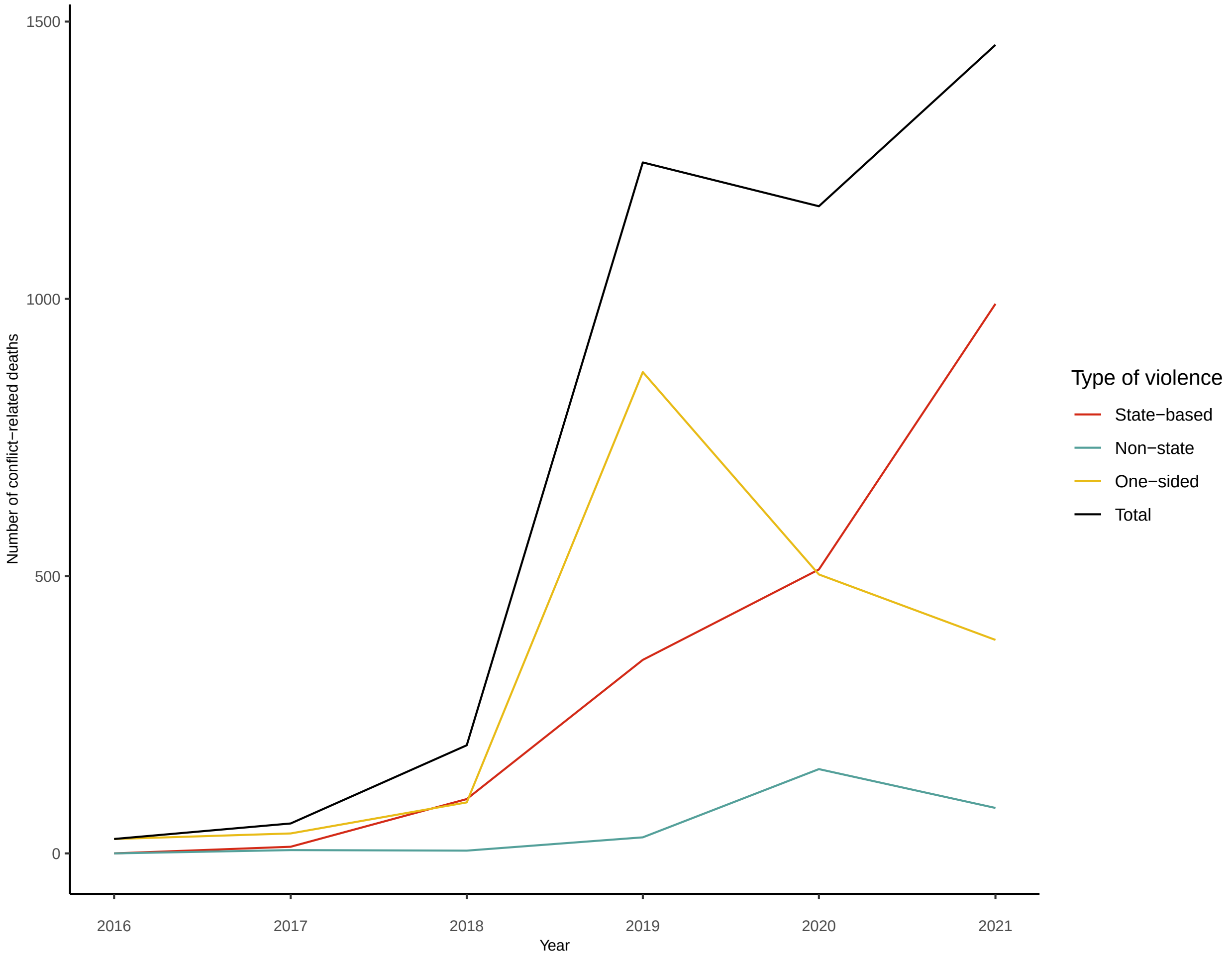

Figure S4: Conflict-related deaths by type of violence over time, 2016–2021

The figure shows the UCDP conflict-related deaths over time from 2016 to 2021, total and by type of violence. In our analysis, we used conflict events of any type of violence – State-based: The use of armed force between two parties, of which at least one is the government of a state; Non-state: The use of armed force between two organised armed groups, neither of which is the government of a state; One-sided: The use of armed force by the government of a state or by a formally organised group against civilians.

5. Distribution of CSPS and CM facilities (left) and conflict events (right) in Burkina Faso, 2016–2021

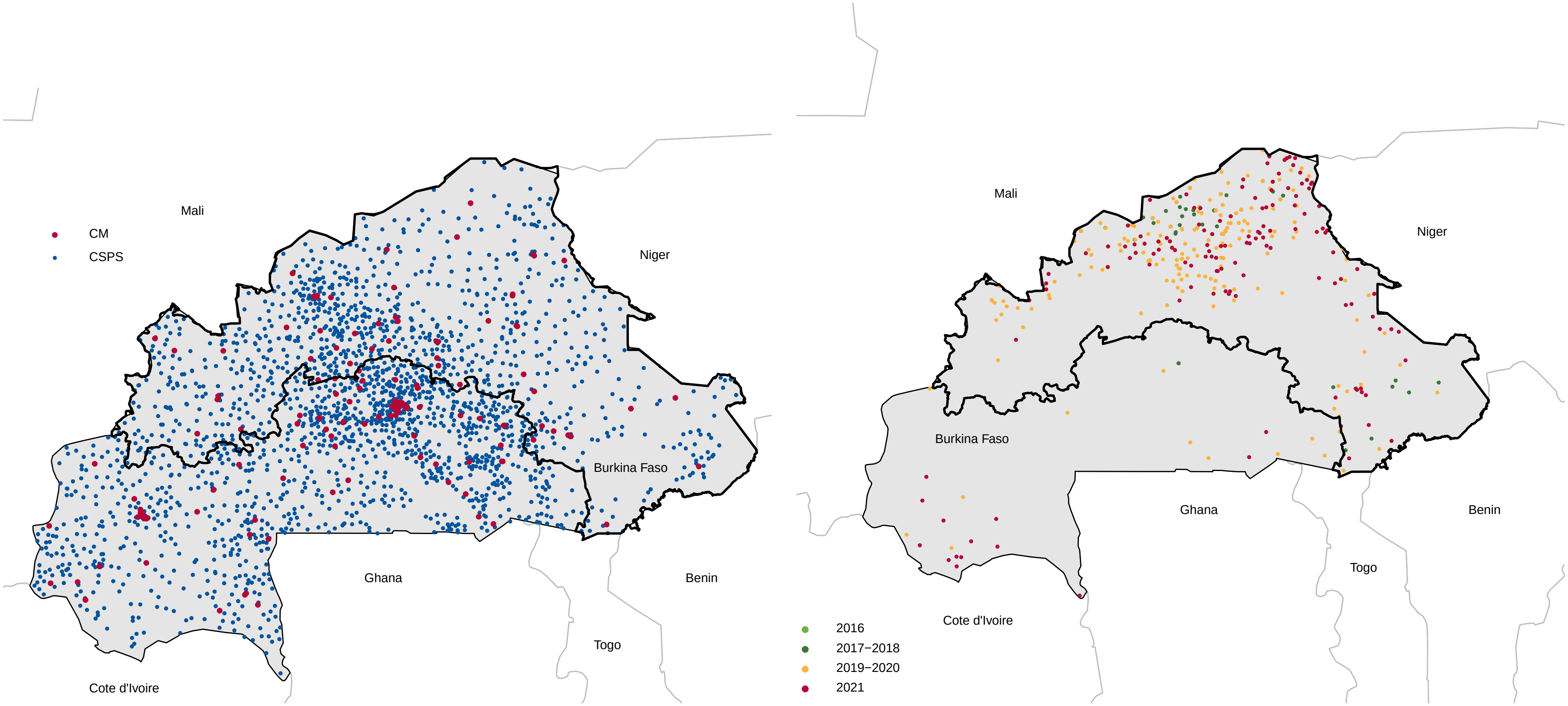

Figure S5: Distribution of CSPS and CM facilities (left) and conflict events (right) in Burkina Faso, 2016–2021

The left map shows all CSPS (blue, n=2105) and CM (red, n=199) facilities in Burkina Faso. The right map shows the Uppsala Conflict Data Program conflict location data in Burkina Faso, with changing location over time from 2016 to 2021. The study country Burkina Faso is shown in grey. The area examined in the geospatial analyses is displayed with thick borders.

6. Distribution of CSPS and CM facilities (left) and conflict events (right) in Burkina Faso, 2016–2021

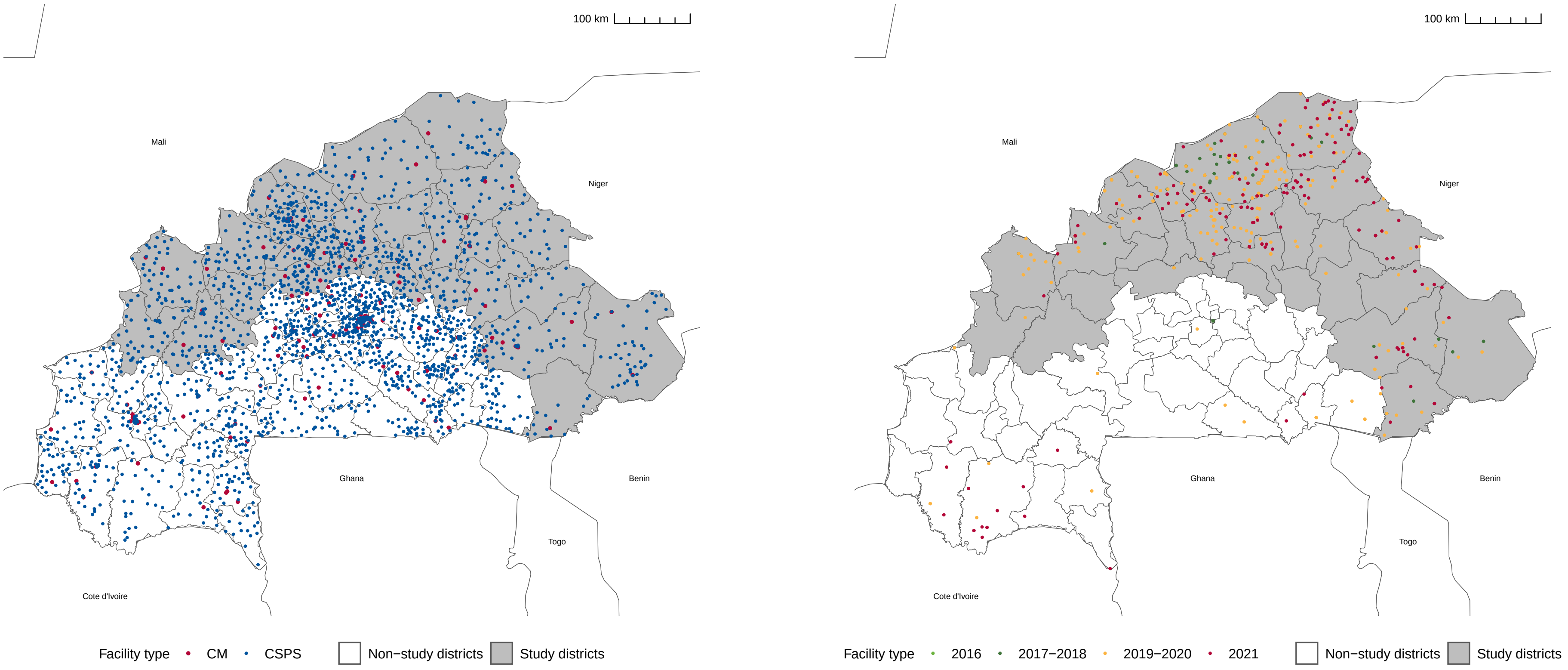

Figure S6: Distribution of CSPS and CM facilities (left) and conflict events (right) in Burkina Faso, 2016–2021

The left map shows all CSPS (blue, n=2105) and CM (red, n=199) facilities in Burkina Faso. The right map shows the Uppsala Conflict Data Program conflict location data in Burkina Faso, with changing location over time from 2016 to 2021. The study country Burkina Faso is shown in grey. The area examined in the geospatial analyses is displayed with thick borders.

7. Trend in the interquartile range of facility-based deliveries

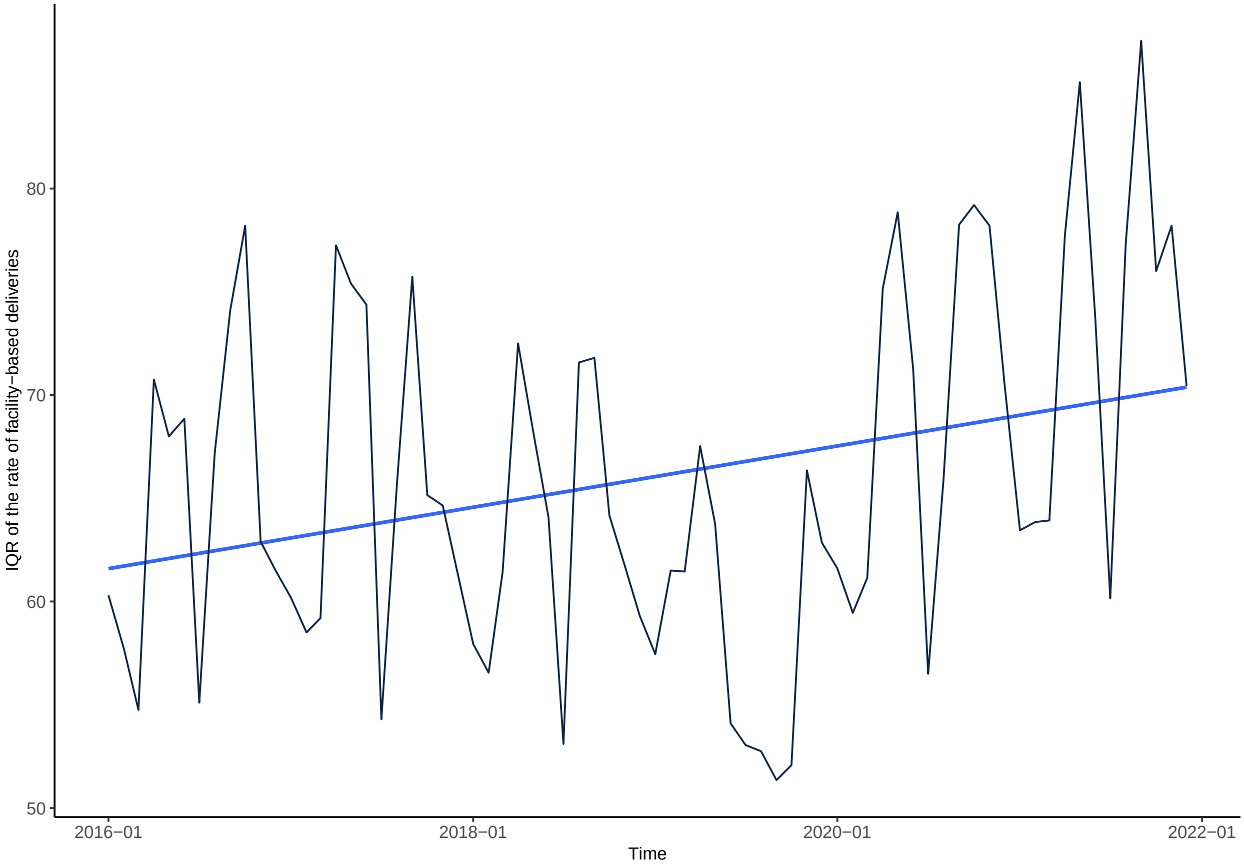

Figure S7: Trend in the interquartile range of facility-based deliveries

References

[1] Iglewicz B, Hoaglin DC. Volume 16: How to Detect and Handle Outliers. Milwaukee, MI: American Society for Quality Control; 1993.

[2] Pérez-González A, Vilar-Fernández JM, González-Manteiga W. Asymptotic properties of local polynomial regression with missing data and correlated errors. Annals of the Institute of Statistical Mathematics. 2009 Mar;61(1):85-109. Available from: https://doi.org/10.1007/s10463-007-0136-2.

[3] You W, Evangelou IE, Zun Z, Andescavage N, Limperopoulos C. Robust preprocessing for stimulus-based functional MRI of the moving fetus. Journal of Medical Imaging. 2016 Apr;3(2):026001. Available from: https://www.ncbi.nlm.nih.gov/pmc/articles/PMC4821892/.

[4] Horton NJ, Kleinman KP. Much ado about nothing: a comparison of missing data methods and software to fit incomplete data regression models. The American Statistician. 2007 Feb;61(1):79-90.

[5] Honaker J, King G. What to do About Missing Values in Time Series Cross-Section Data. American Journal of Political Science. 2010;54(3):561-81.

[6] Dong Y, Peng CYJ. Principled missing data methods for researchers. SpringerPlus. 2013 May;2(1):222. Available from: https://doi.org/10.1186/2193-1801-2-222.

[7] Pratama I, Permanasari AE, Ardiyanto I, Indrayani R. A review of missing values handling methods on time-series data. 2016 International Conference on Information Technology Systems and Innovation (ICITSI). 2016 Oct:1-6. Conference Name: 2016 International Conference on Information Technology Systems and Innovation (ICITSI) ISBN: 9781509024490 Place: Bandung - Bali, Indonesia Publisher: IEEE. Available from: http://ieeexplore.ieee.org/document/7858189/.

[8] Kuunibe N, Lohmann J, Hillebrecht M, Nguyen HT, Tougri G, De Allegri M. What happens when performance-based financing meets free healthcare? Evidence from an interrupted time-series analysis. Health Policy and Planning. 2020 Oct;35(8):906-17.

[9] WorldPop, Institut National de la Statistique et de la Démographie du Burkina Faso. Census-based gridded population estimates for Burkina Faso (2019), version 1.1. University of Southampton: WorldPop; 2022. Doi: 10.5258/SOTON/WP00736. Available from: https://tiles.arcgis.com/tiles/BU6Aadhn6tbBEdyk/arcgis/rest/services/bfa_population_v1_1_tile/MapServer.
