# Supplementary material for: The evolution of facility-based deliveries at primary healthcare centres during an insecurity and conflict crisis in Burkina Faso: a geospatial analysis
Source: Confl Health. 2025 Nov 3;19:78. doi: 10.1186/s13031-025-00723-8 (PMC12581450; doi:10.1186/s13031-025-00723-8)
Supplement: Supplementary file 2 — Supplementary material file 2 [file 13031_2025_723_MOESM2_ESM.pdf]

Fig. 2 – 2016

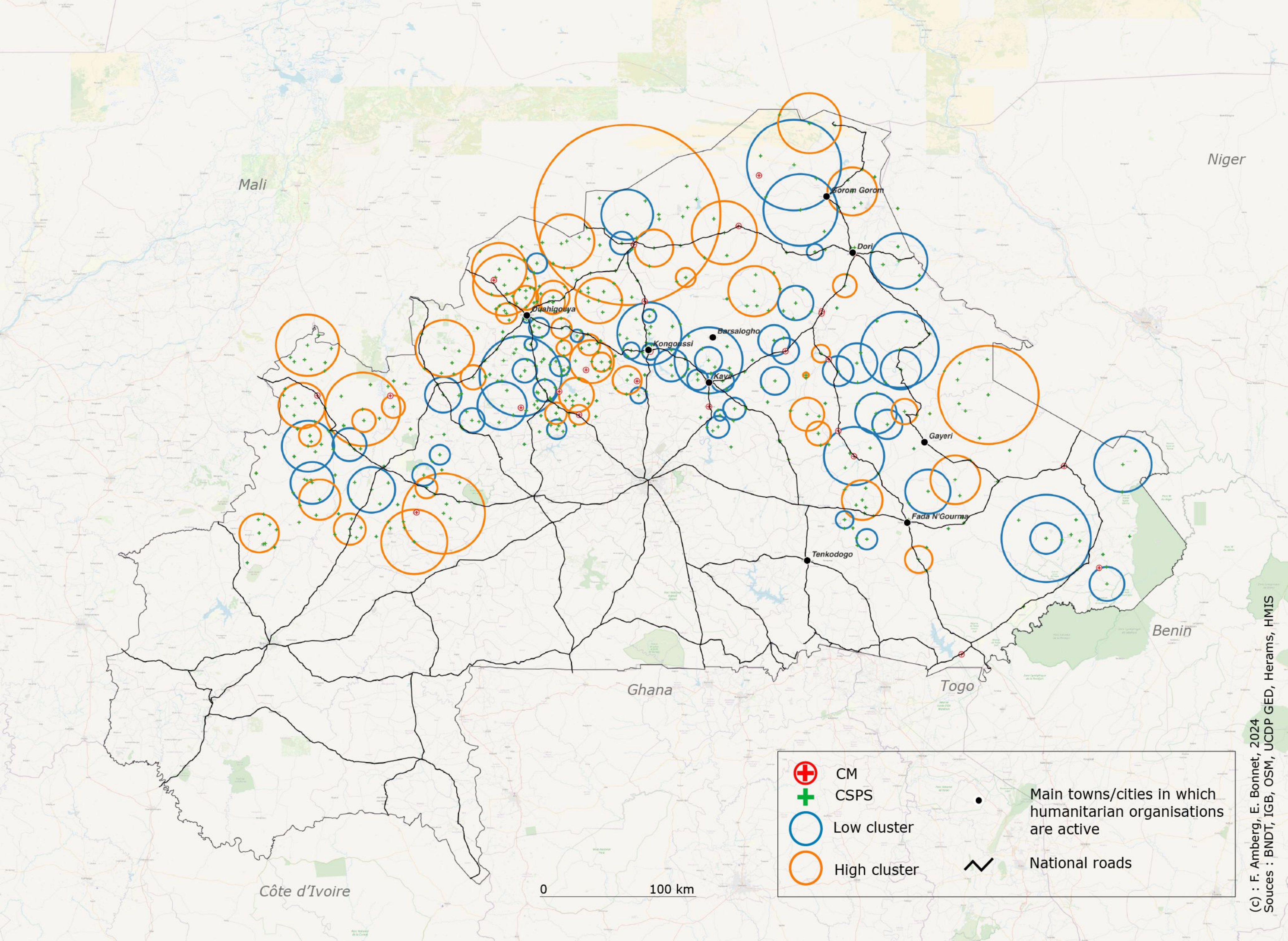
Mali
Niger
Benin
Togo
Ghana
Côte d'Ivoire
Gorom Gorom
Dori
Ouahigouya
Barsalogho
Kongoussi
Kaya
Gayeri
Fada N'Gourma
Tenkodogo
0
100 km
CM
CSPS
Low cluster
High cluster
Main towns/cities in which humanitarian organisations are active
National roads
(c) : F. Amberg, E. Bonnet, 2024
Souces : BNDT, IGB, OSM, UCDP GED, Herams, HMIS

Fig. 2 – 2017

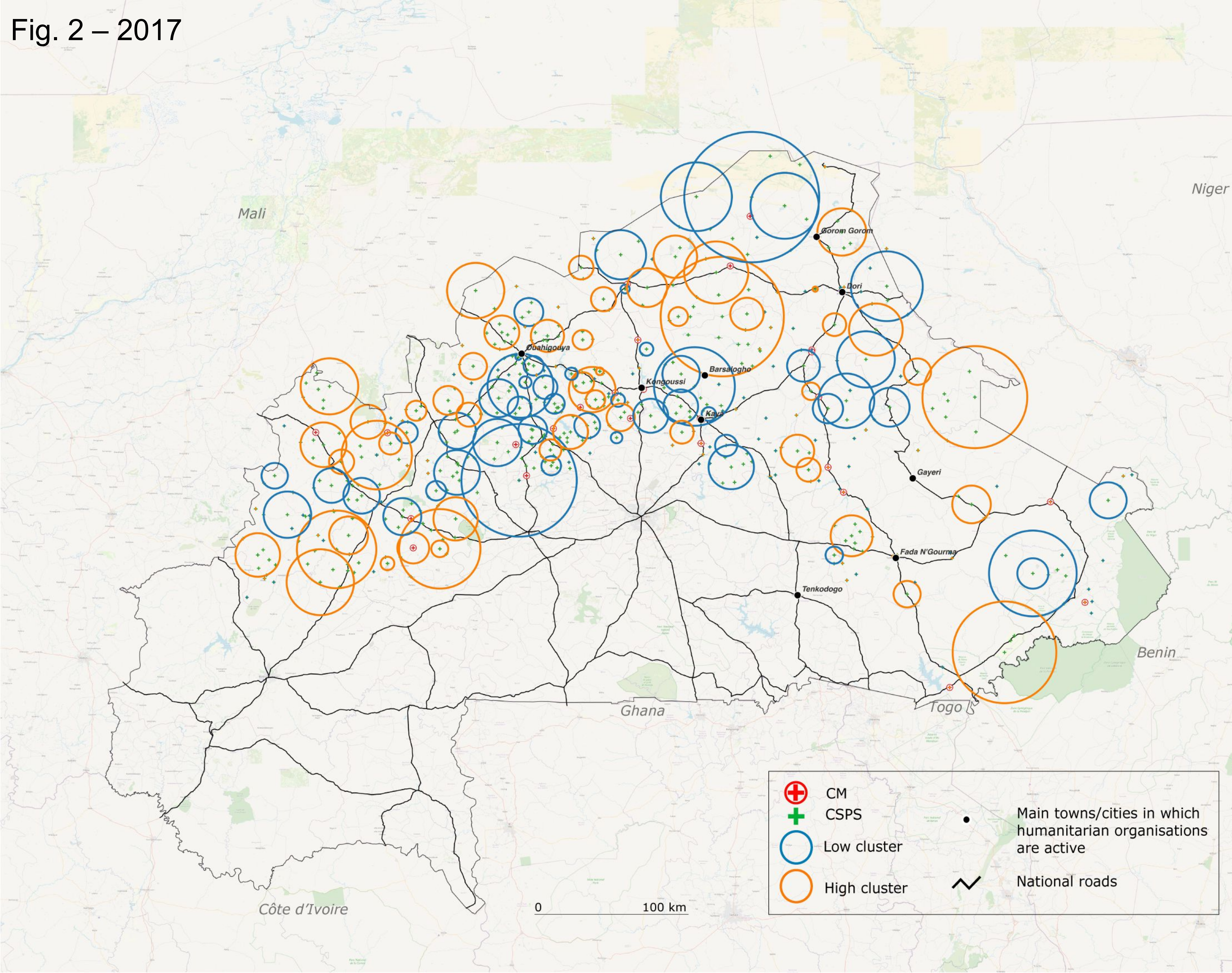

(c) : F. Amberg, E. Bonnet, 2024
Souces : BNDT, IGB, OSM, UCDP GED, Herams, HMIS

Fig. 2 – 2018

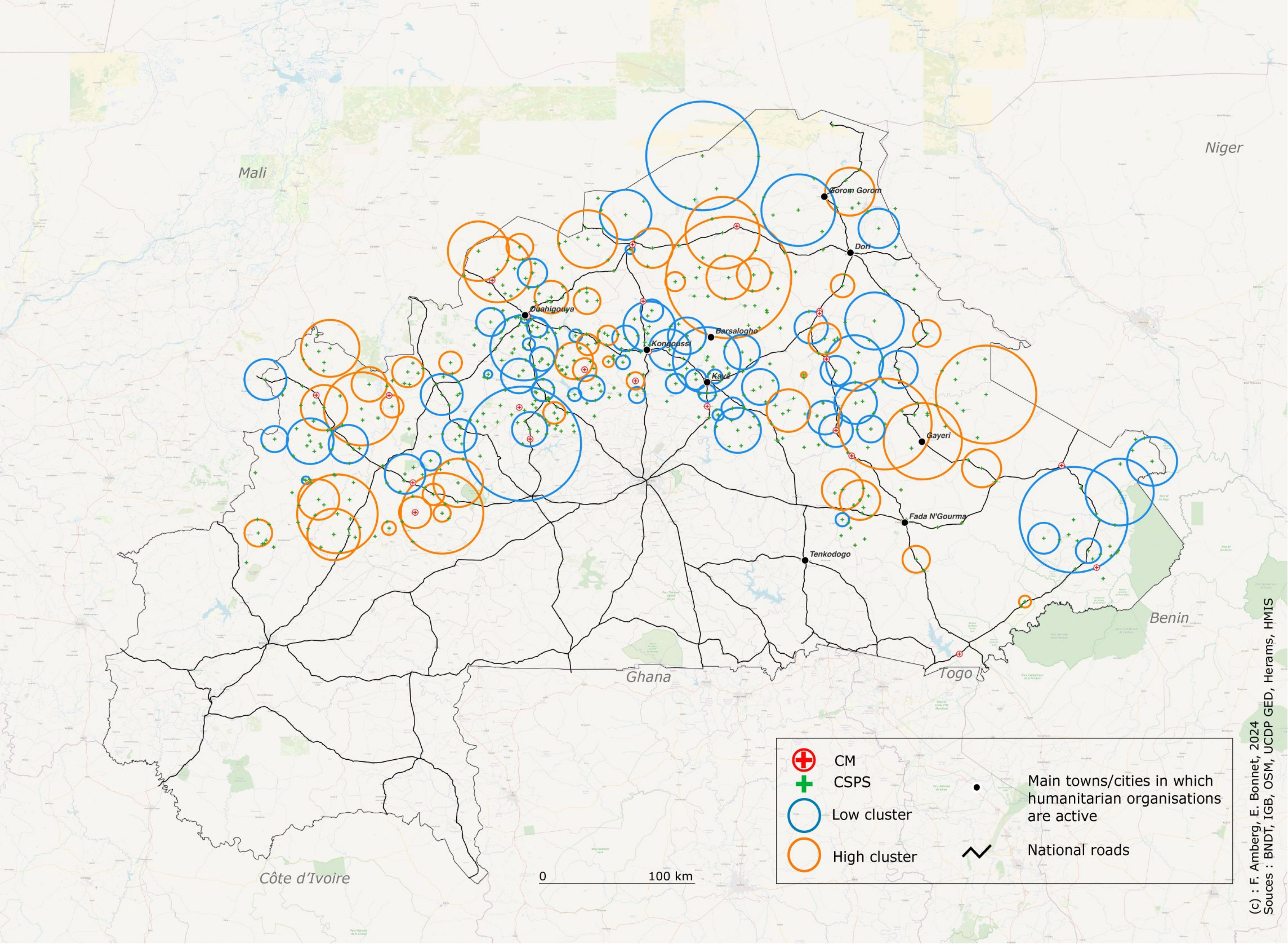
Mali
Niger
Benin
Togo
Ghana
Côte d'Ivoire
Gorom Gorom
Dori
Ouahigouya
Barsalogho
Kongoussi
Kaya
Gayeri
Fada N'Gourma
Tenkodogo
CM
CSPS
Low cluster
High cluster
Main towns/cities in which humanitarian organisations are active
National roads
0
100 km
(c) : F. Amberg, E. Bonnet, 2024
Souces : BNDT, IGB, OSM, UCDP GED, Herams, HMIS

Fig. 2 – 2019

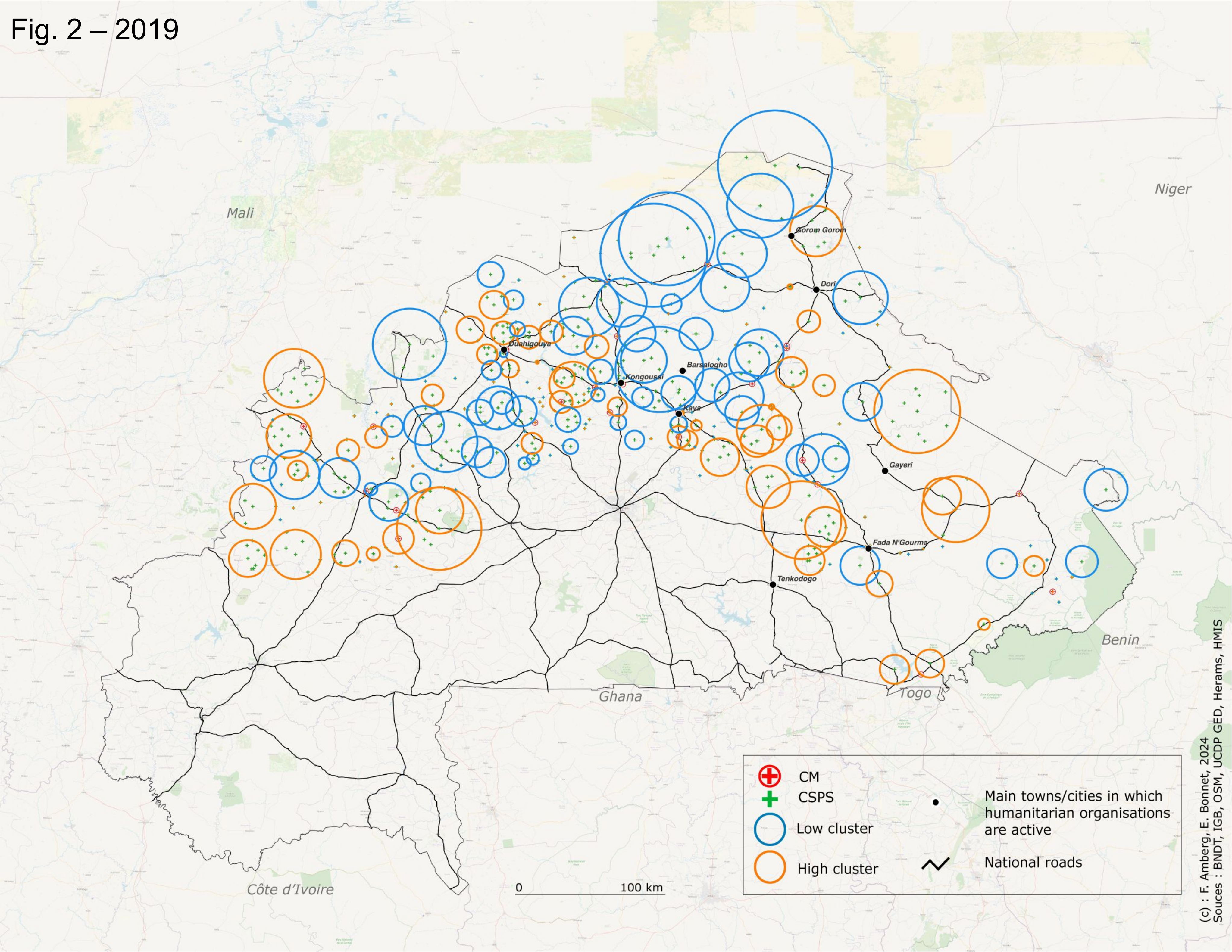
Mali
Niger
Benin
Togo
Ghana
Côte d'Ivoire
Gorom Gorom
Dori
Ouahigouya
Barsalogho
Kongoussi
Kaya
Gayeri
Fada N'Gourma
Tenkodogo
CM
CSPS
Low cluster
High cluster
Main towns/cities in which humanitarian organisations are active
National roads
0
100 km
(c) : F. Amberg, E. Bonnet, 2024
Souces : BNDT, IGB, OSM, UCDP GED, Herams, HMIS

Fig. 2 – 2020

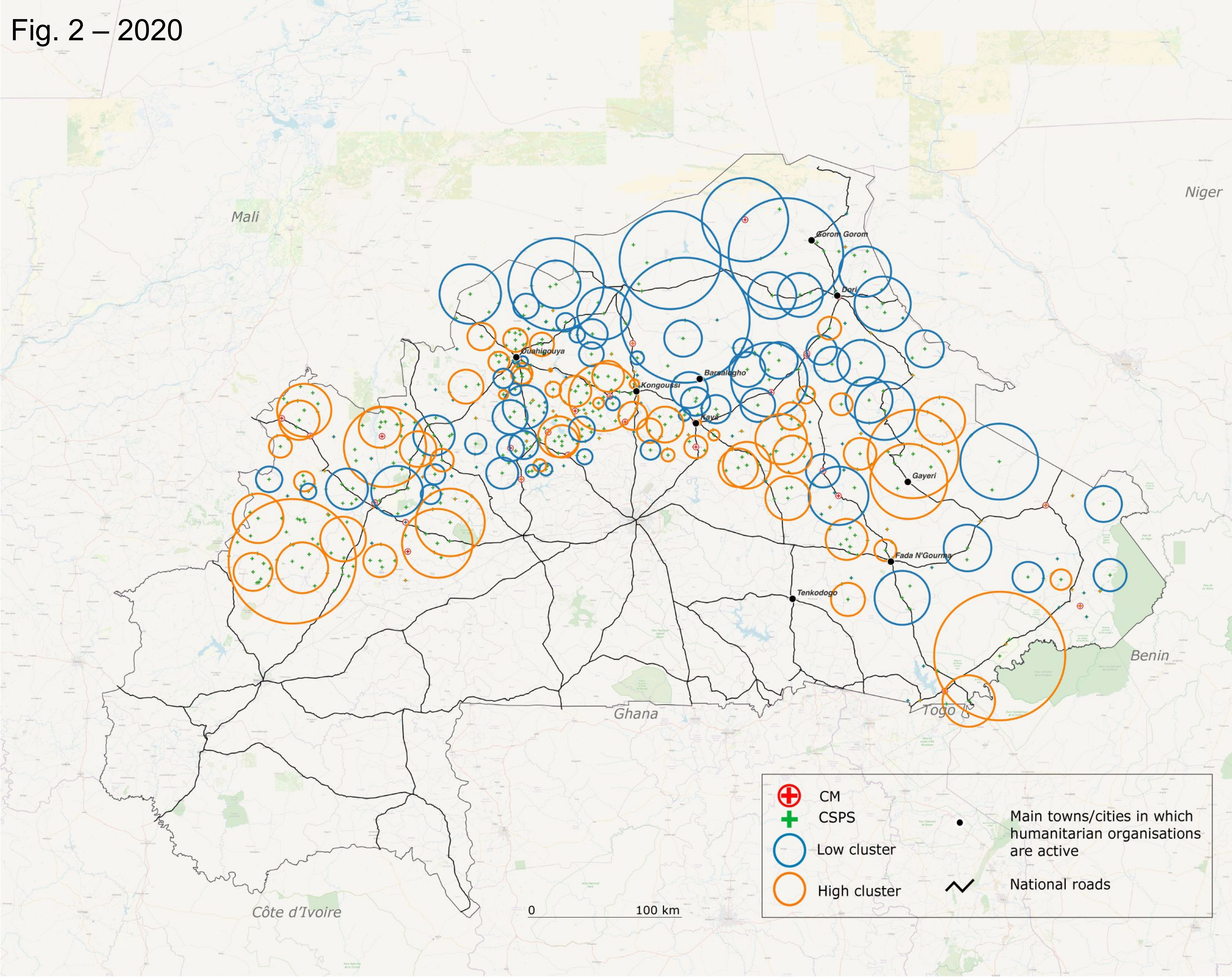

(c) : F. Amberg, E. Bonnet, 2024
Souces : BNDT, IGB, OSM, UCDP GED, Herams, HMIS

Fig. 2 – 2021

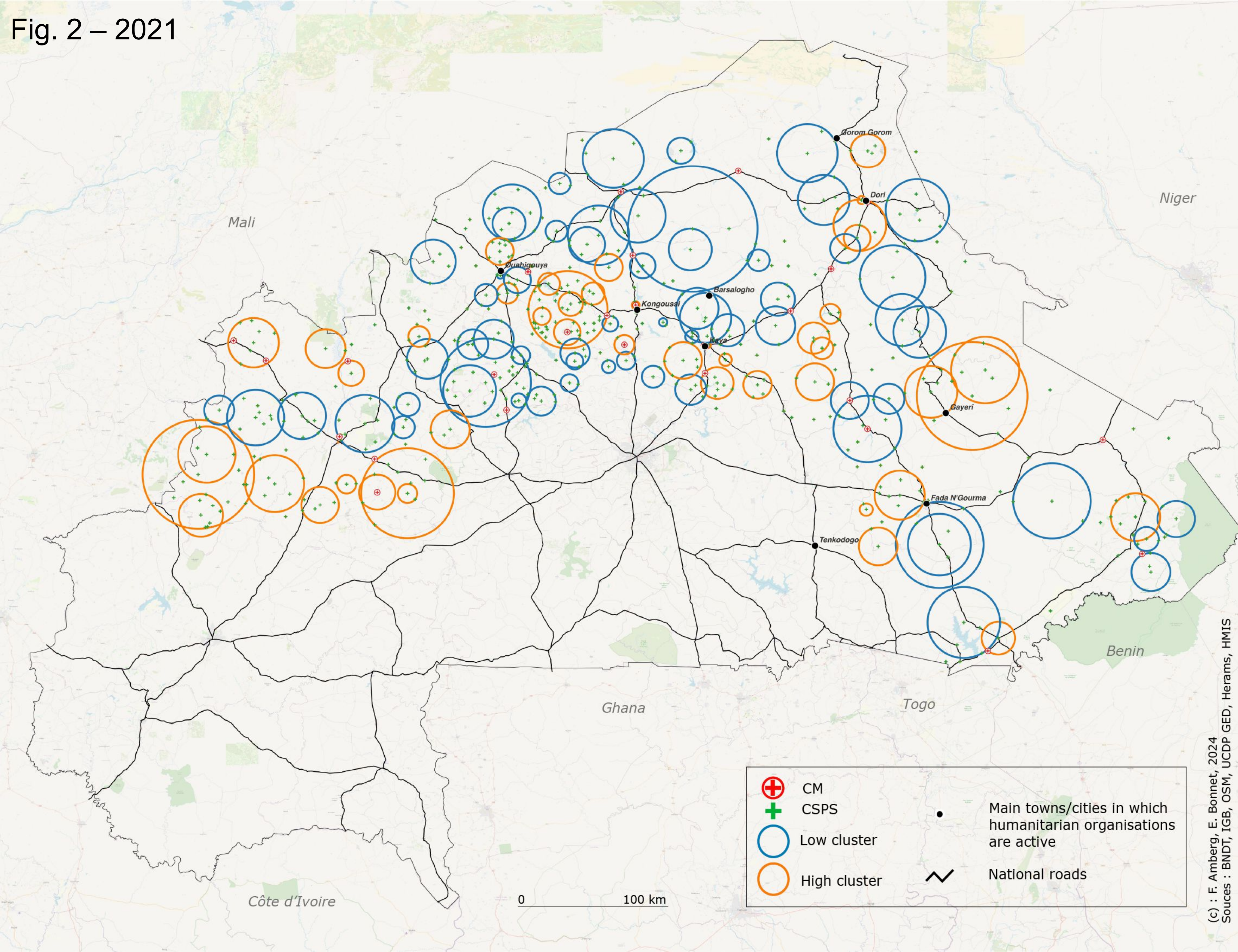
Mali
Niger
Benin
Togo
Ghana
Côte d'Ivoire
Gorom Gorom
Dori
Ouahigouya
Kongoussi
Barsalogho
Kaya
Gayeri
Fada N'Gourma
Tenkodogo
CM
CSPS
Low cluster
High cluster
Main towns/cities in which humanitarian organisations are active
National roads
0
100 km
(c) : F. Amberg, E. Bonnet, 2024
Souces : BNDT, IGB, OSM, UCDP GED, Herams, HMIS

Fig. 3 – 2017

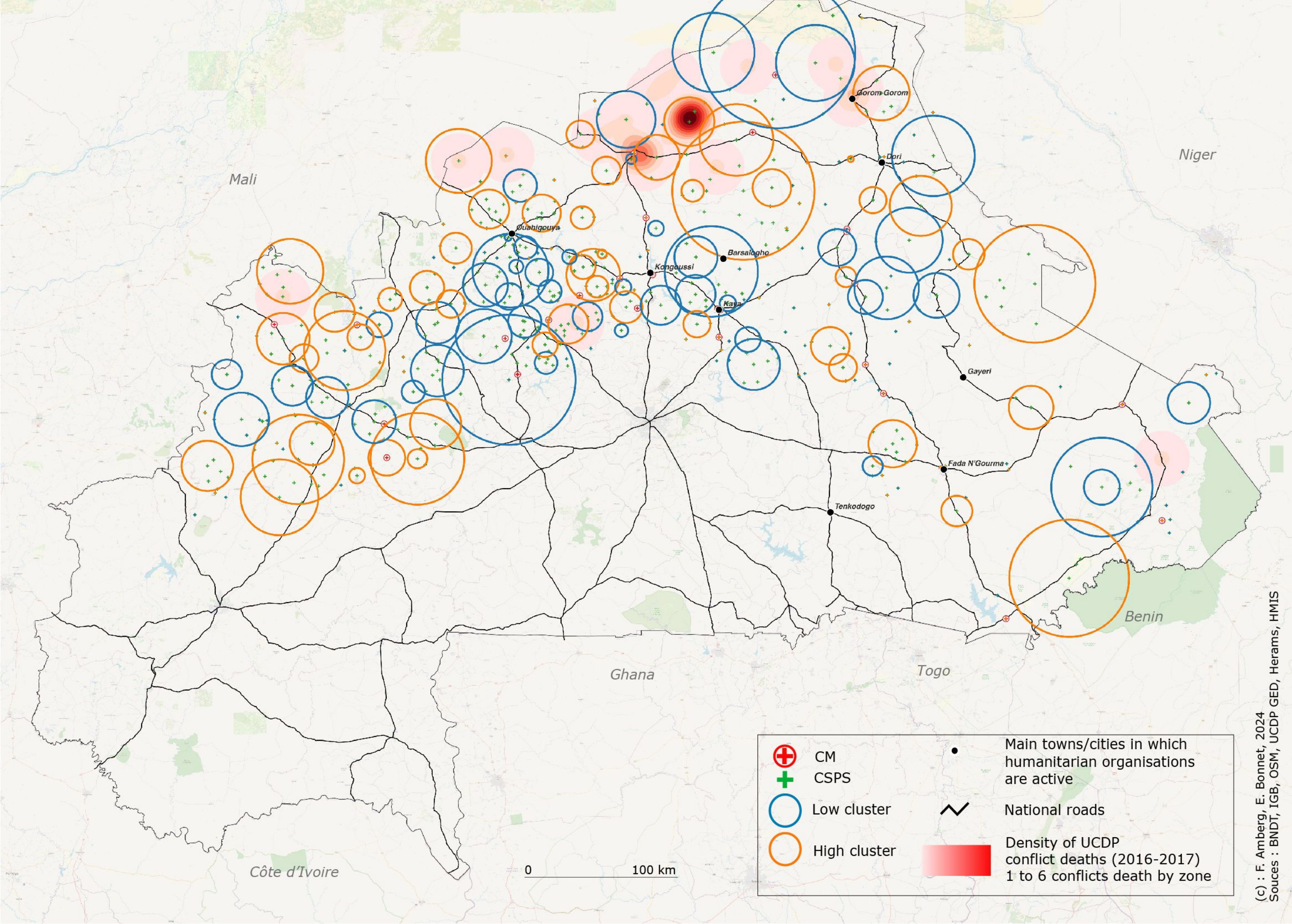

Mali
Niger
Benin
Ghana
Togo
Côte d'Ivoire
Gorom-Gorom
Dori
Ouahigouya
Barsalogho
Kongoussi
Kaya
Gayeri
Fada N'Gourma
Tenkodogo
CM
CSPS
Low cluster
High cluster
Main towns/cities in which humanitarian organisations are active
National roads
Density of UCDP conflict deaths (2016-2017)
1 to 6 conflicts death by zone
0
100 km
(c) : F. Amberg, E. Bonnet, 2024
Souces : BNDT, IGB, OSM, UCDP GED, Herams, HMIS

Fig. 3 – 2018

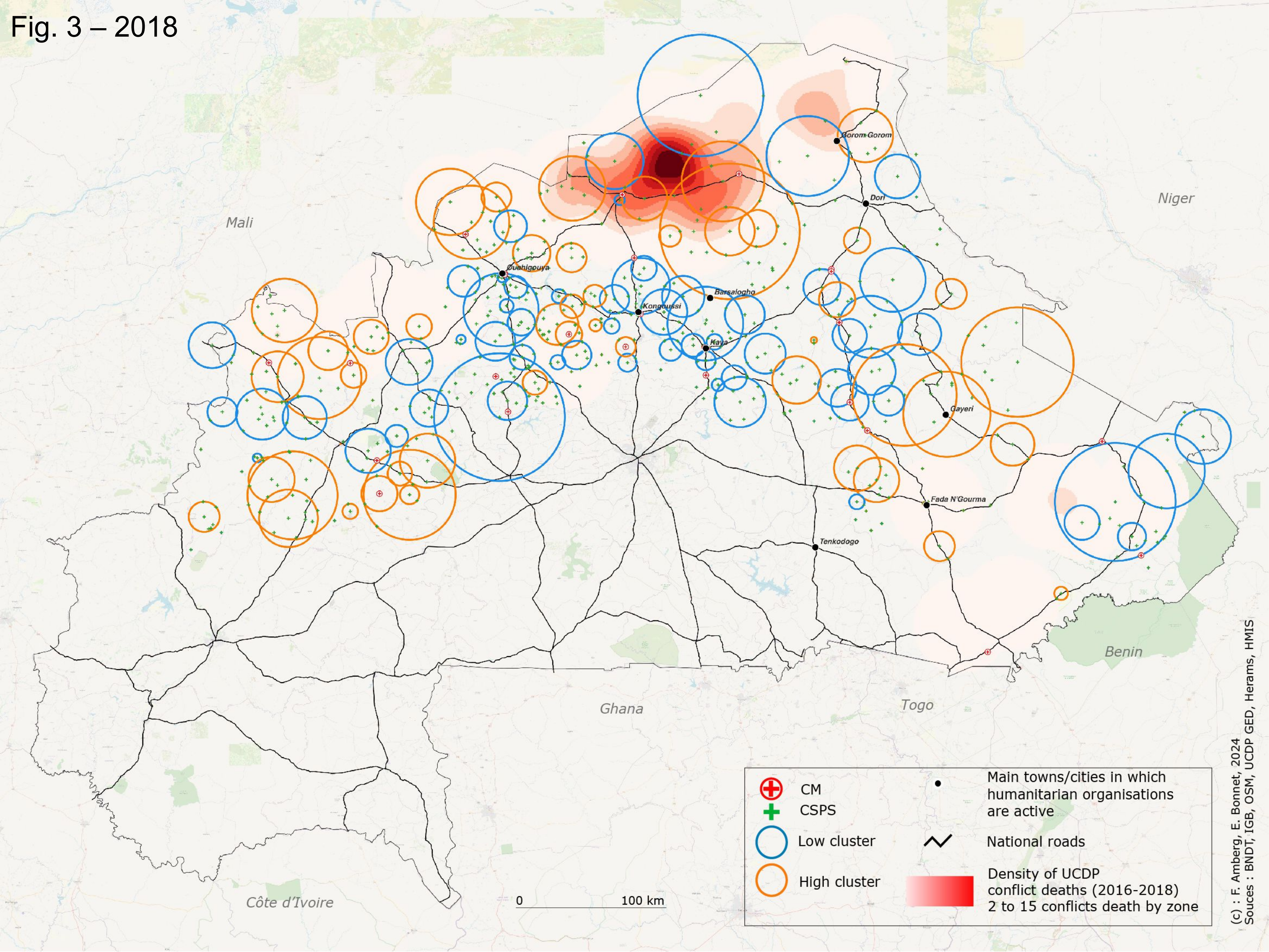
Mali
Niger
Benin
Ghana
Togo
Côte d'Ivoire
Gorom Gorom
Dori
Ouahigouya
Kongoussi
Barsalogho
Kaya
Gayeri
Fada N'Gourma
Tenkodogo
CM
CSPS
Low cluster
High cluster
Main towns/cities in which humanitarian organisations are active
National roads
Density of UCDP conflict deaths (2016-2018) 2 to 15 conflicts death by zone
0
100 km
(c) : F. Amberg, E. Bonnet, 2024
Souces : BNDT, IGB, OSM, UCDP GED, Herams, HMIS

Fig. 3 – 2019

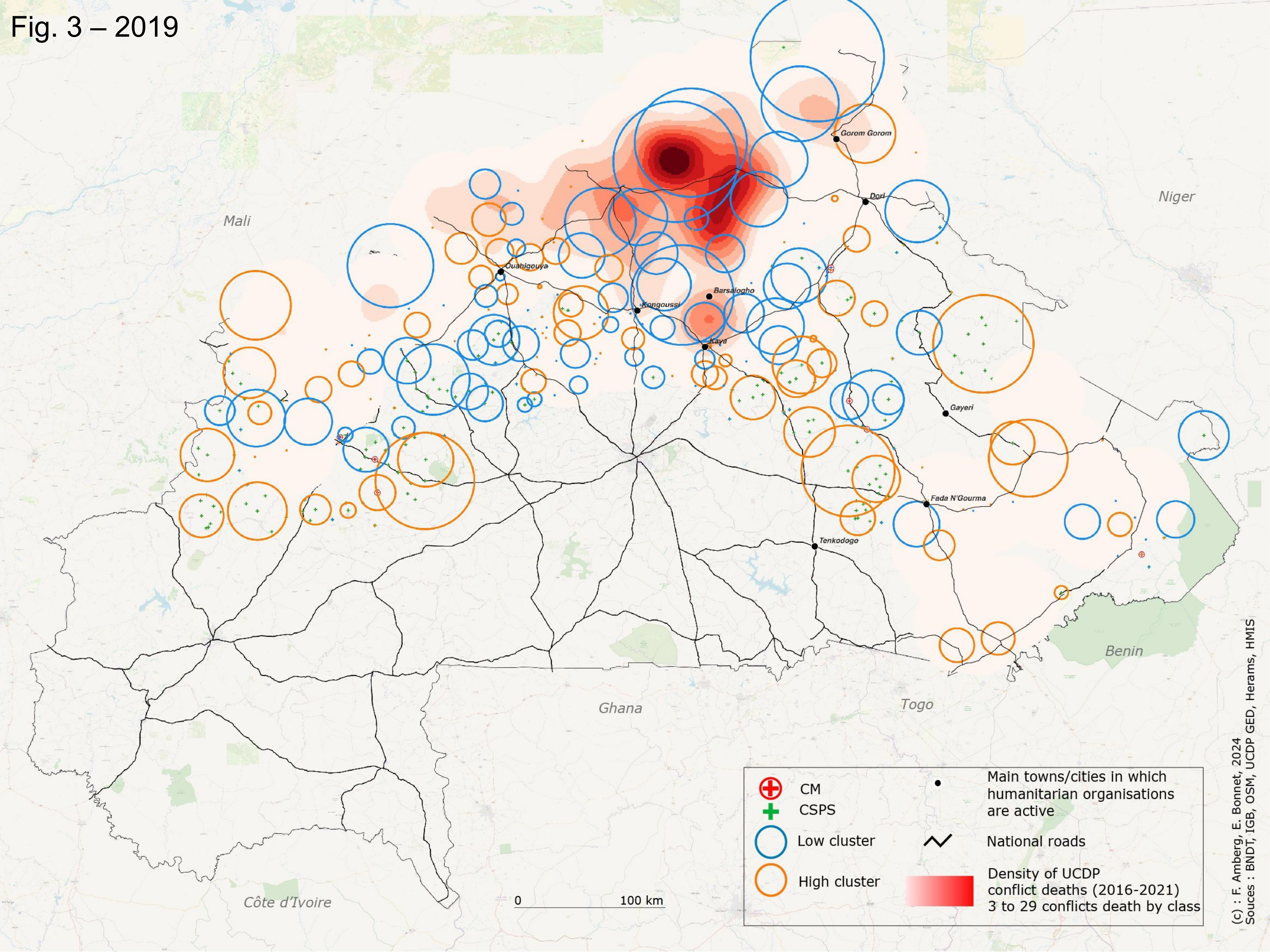

Mali
Niger
Gorom Gorom
Dori
Ouahigouya
Barsalogho
Kongoussi
Kaya
Gayeri
Fada N'Gourma
Tenkodogo
Benin
Togo
Ghana
Côte d'Ivoire
0
100 km
CM
CSPS
Low cluster
High cluster
Main towns/cities in which humanitarian organisations are active
National roads
Density of UCDP conflict deaths (2016-2021)
3 to 29 conflicts death by class
(c) : F. Amberg, E. Bonnet, 2024
Souces : BNDT, IGB, OSM, UCDP GED, Herams, HMIS

Fig. 3 – 2020

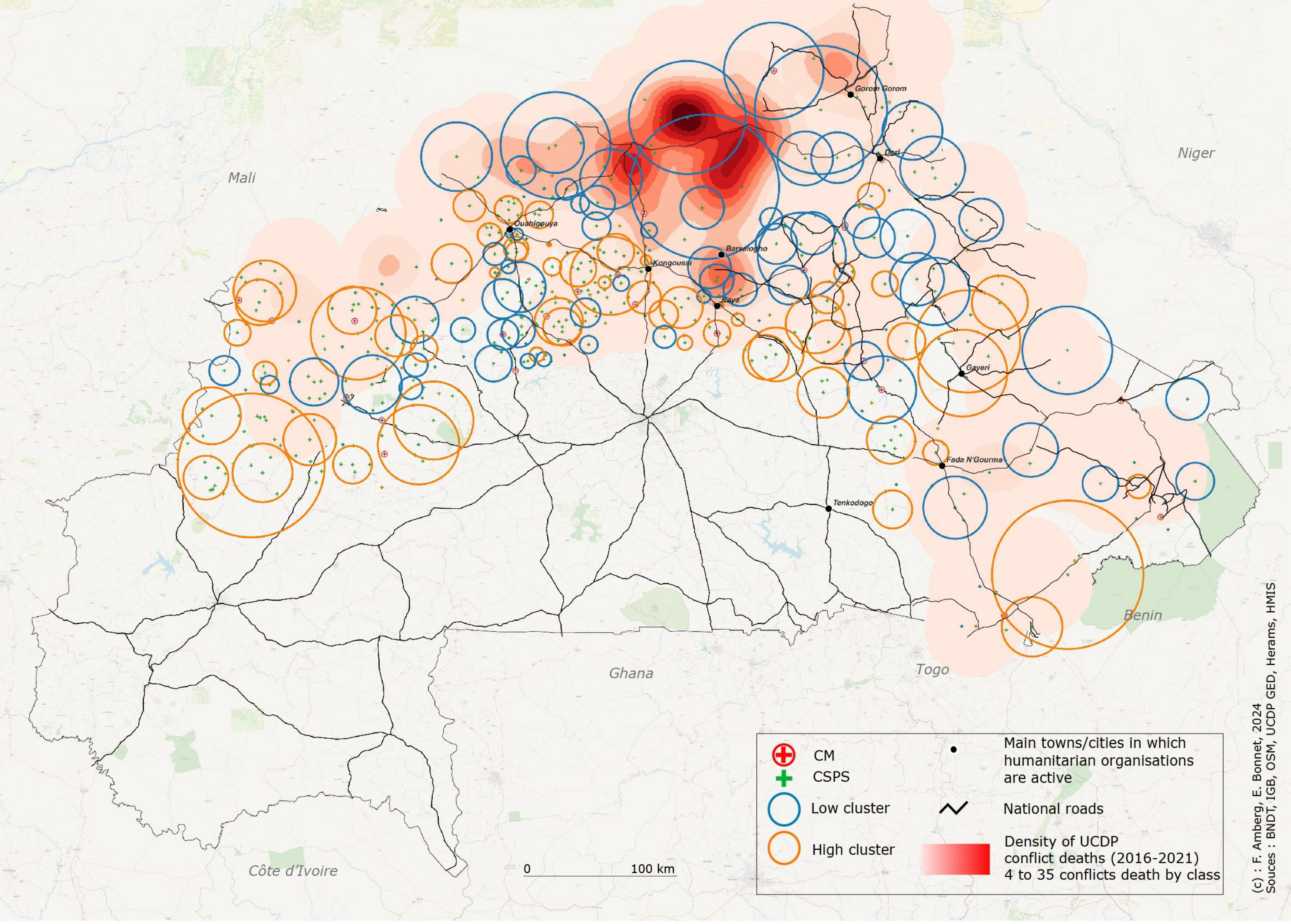
Mali
Niger
Benin
Togo
Ghana
Côte d'Ivoire
Gorom Gorom
Dori
Ouahigouya
Barsalogho
Kongoussi
Kaya
Gayeri
Fada N'Gourma
Tenkodogo
0
100 km
CM
CSPS
Low cluster
High cluster
Main towns/cities in which humanitarian organisations are active
National roads
Density of UCDP conflict deaths (2016-2021) 4 to 35 conflicts death by class
(c) : F. Amberg, E. Bonnet, 2024
Souces : BNDT, IGB, OSM, UCDP GED, Herams, HMIS

Fig. 3 – 2021

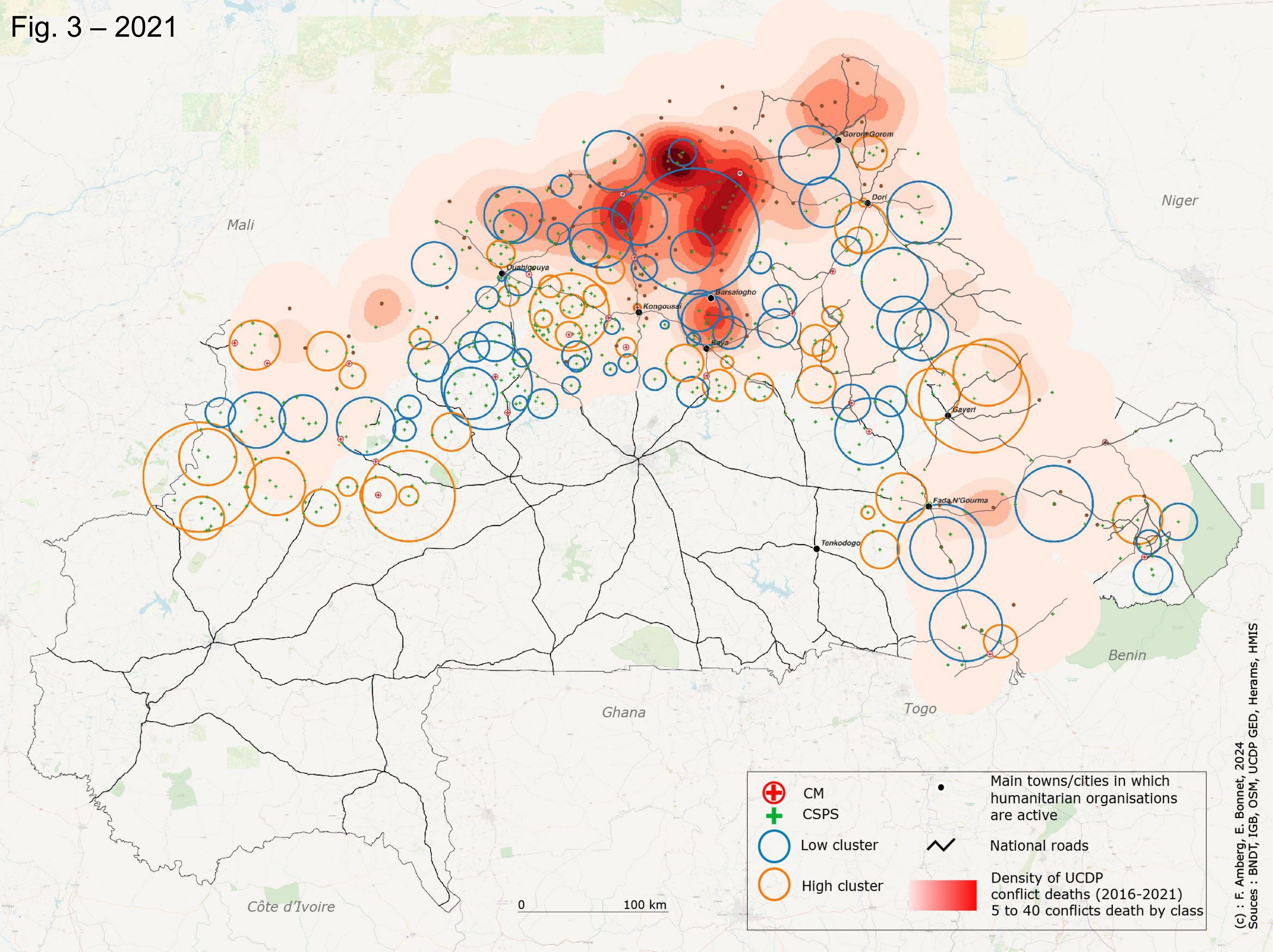
Mali
Niger
Benin
Togo
Ghana
Côte d'Ivoire
Gorom Gorom
Dori
Ouahigouya
Barsalogho
Kongoussi
Kaya
Gayeri
Fada N'Gourma
Tenkodogo
0
100 km
CM
CSPS
Low cluster
High cluster
Main towns/cities in which humanitarian organisations are active
National roads
Density of UCDP conflict deaths (2016-2021) 5 to 40 conflicts death by class
(c) : F. Amberg, E. Bonnet, 2024
Souces : BNDT, IGB, OSM, UCDP GED, Herams, HMIS

Fig. 4 – Facilities with >50 conflict deaths within 25 km (2016–2021)

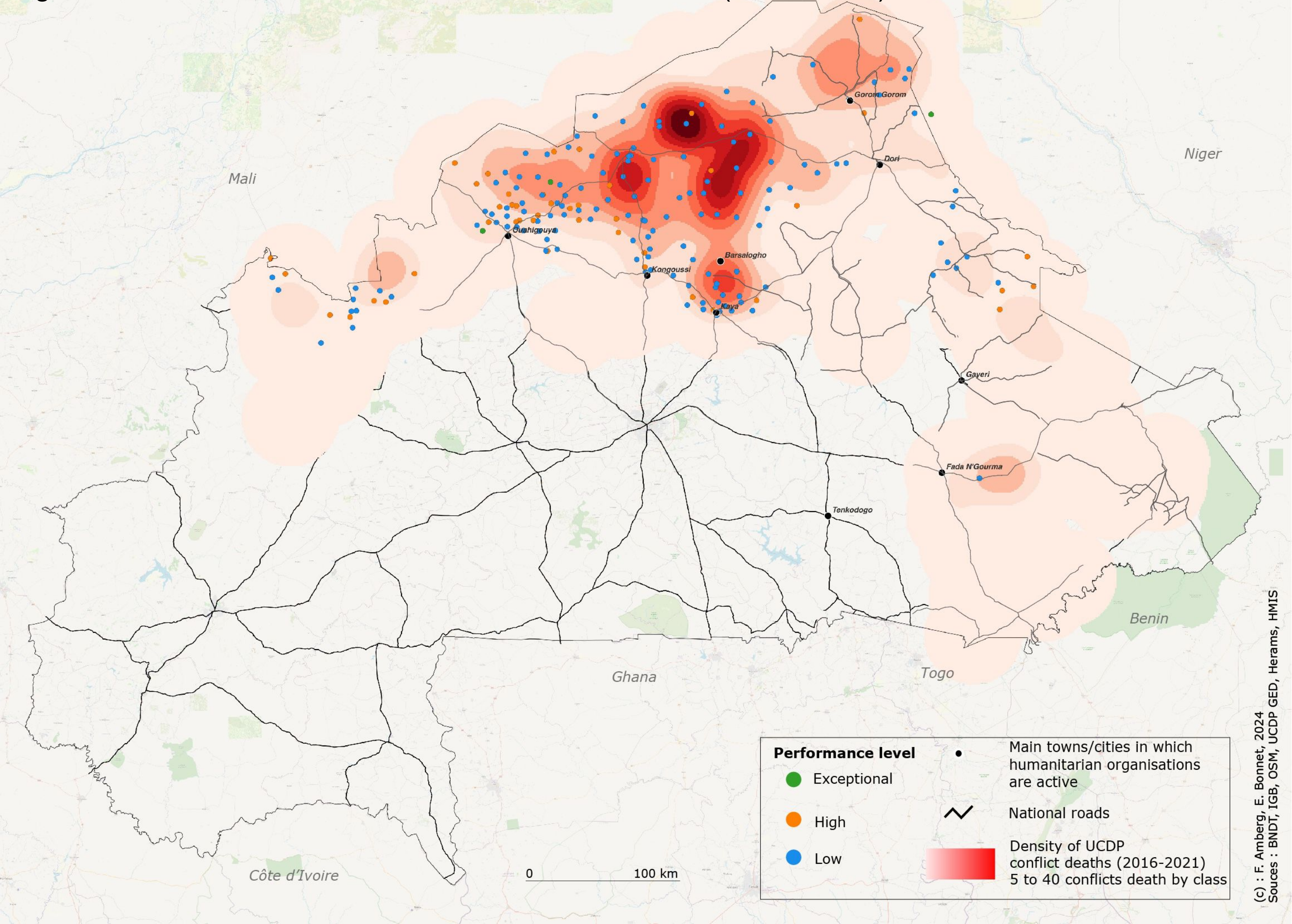

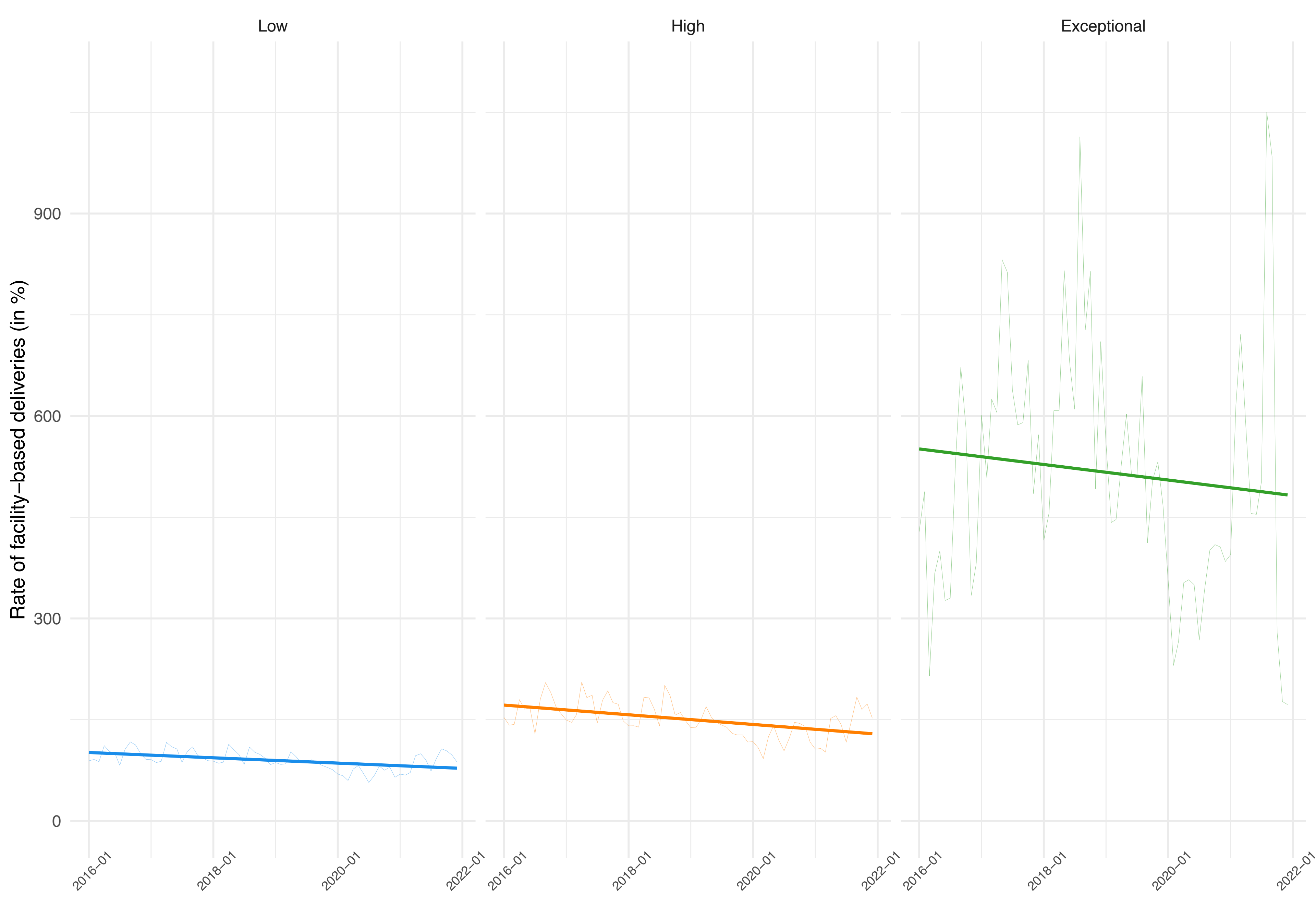
Low
High
Exceptional
Rate of facility–based deliveries (in %)
900
600
300
0
2016–01
2018–01
2020–01
2022–01

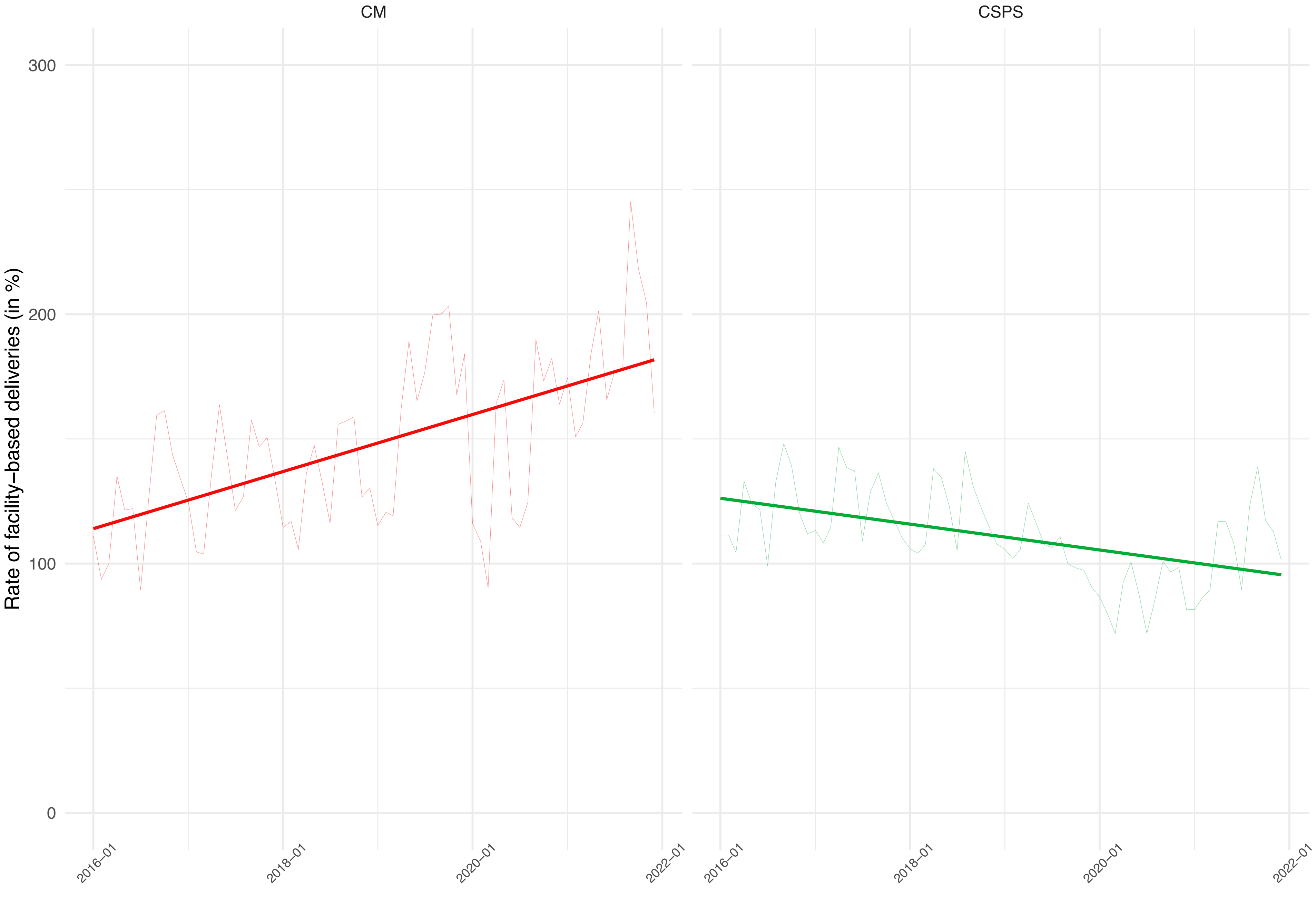

CM
CSPS
Rate of facility–based deliveries (in %)
300
200
100
0
2016–01
2018–01
2020–01
2022–01

Fig. 4 – Facilities with 1–50 conflict deaths within 25 km (2016–2021)

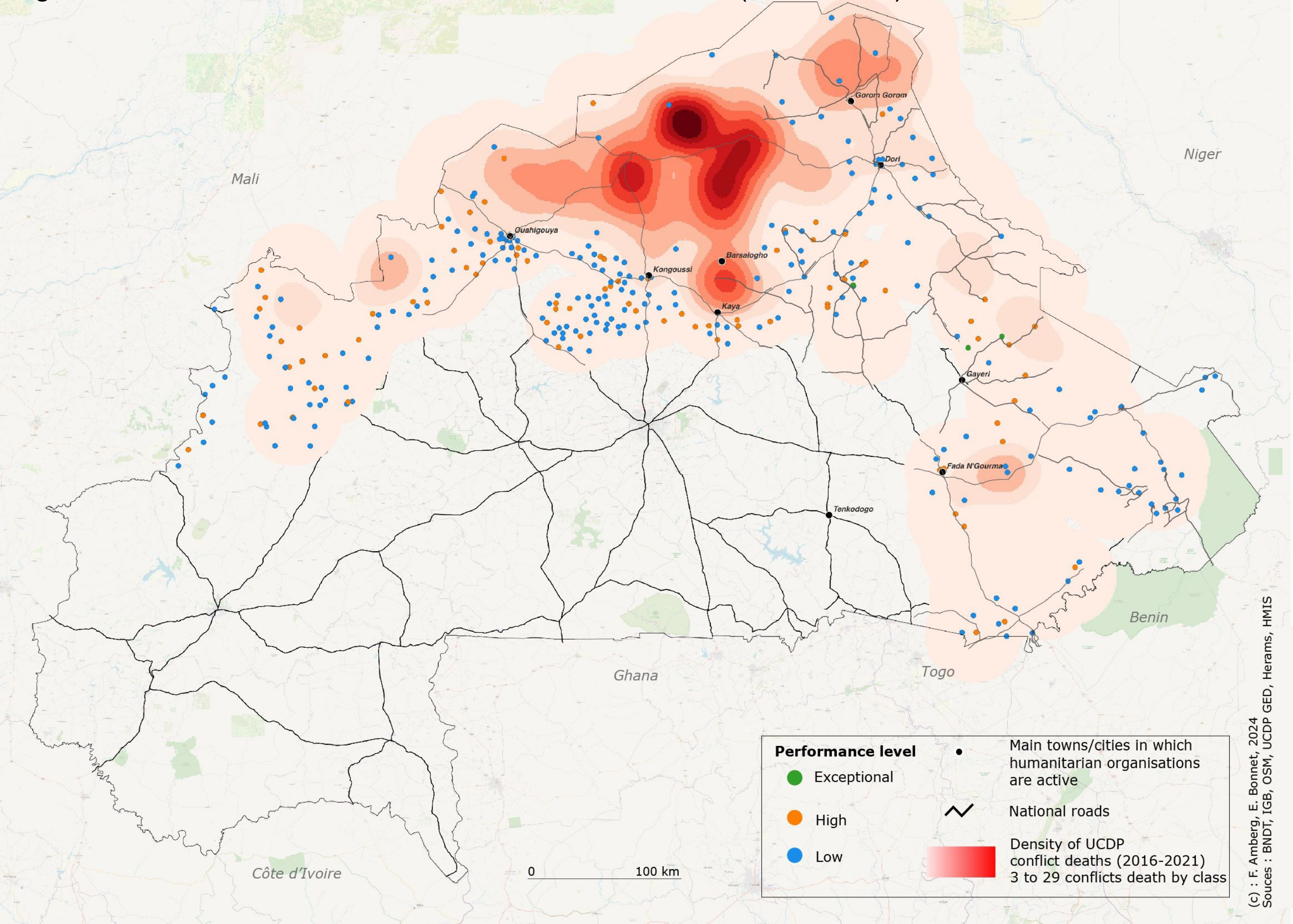

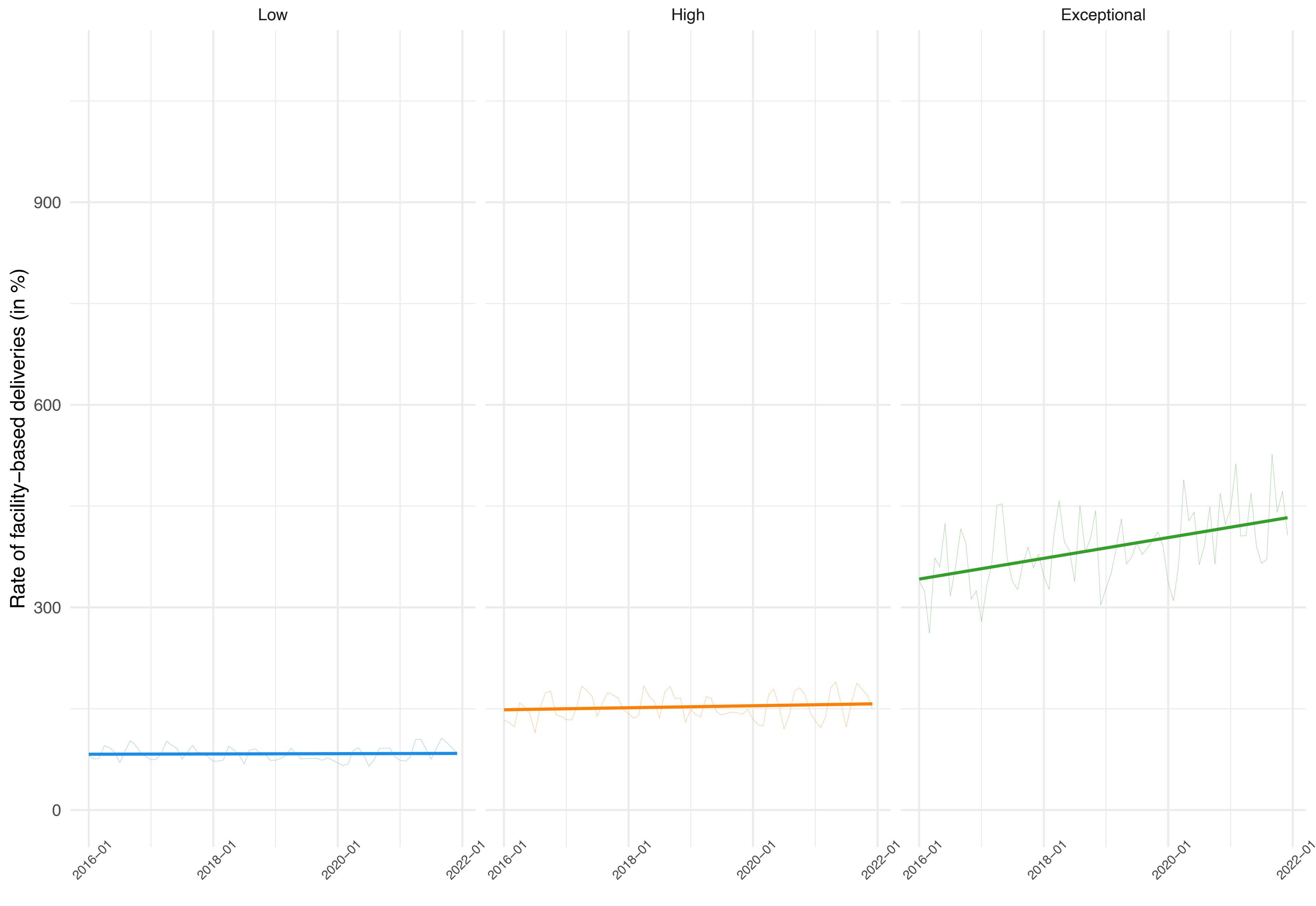
Low
High
Exceptional
Rate of facility−based deliveries (in %)
900
600
300
0
2016−01
2018−01
2020−01
2022−01

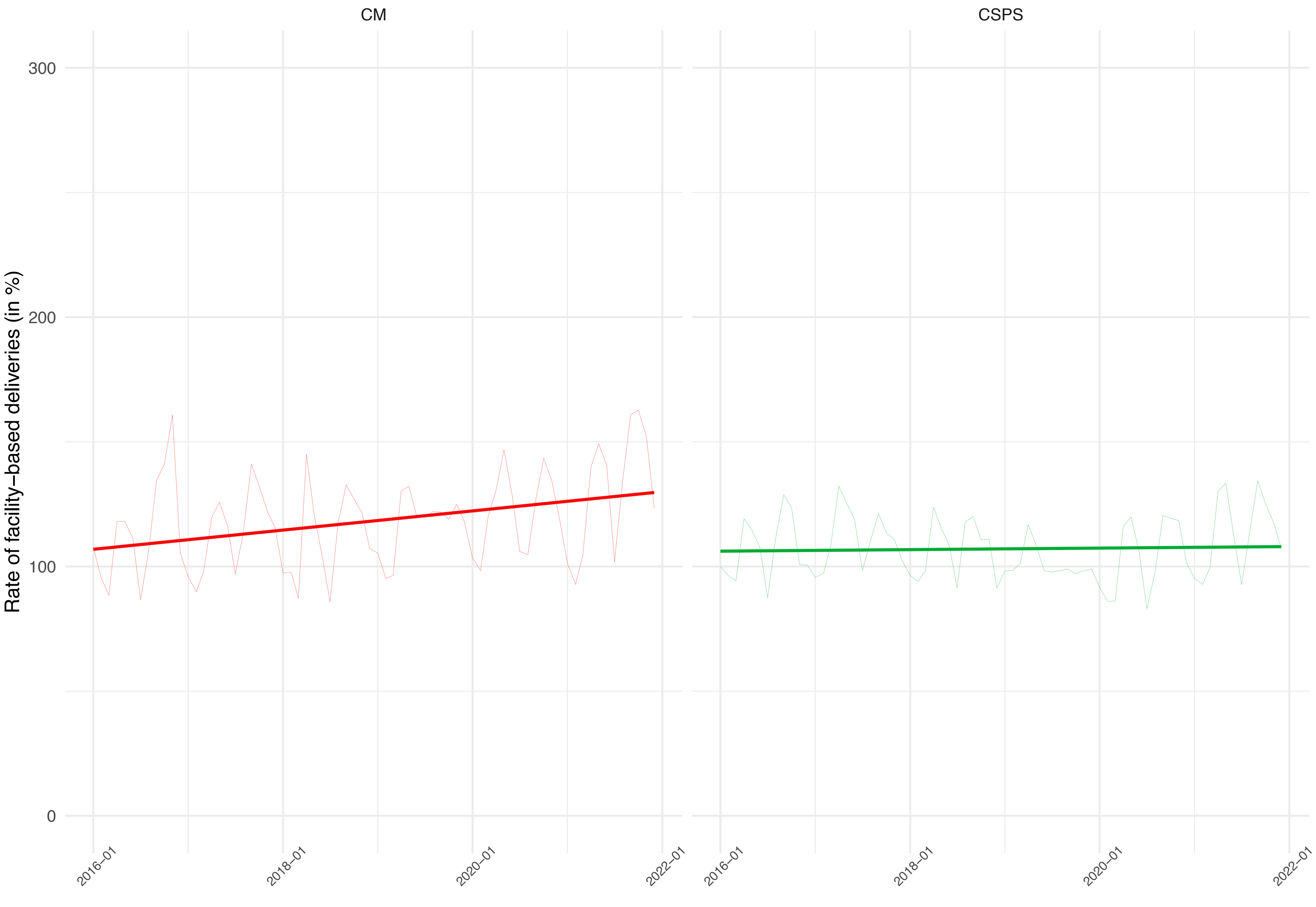
CM
CSPS
Rate of facility–based deliveries (in %)
300
200
100
0
2016–01
2018–01
2020–01
2022–01

Fig. 4 – Facilities with 0 conflict deaths within 25 km (2016–2021)

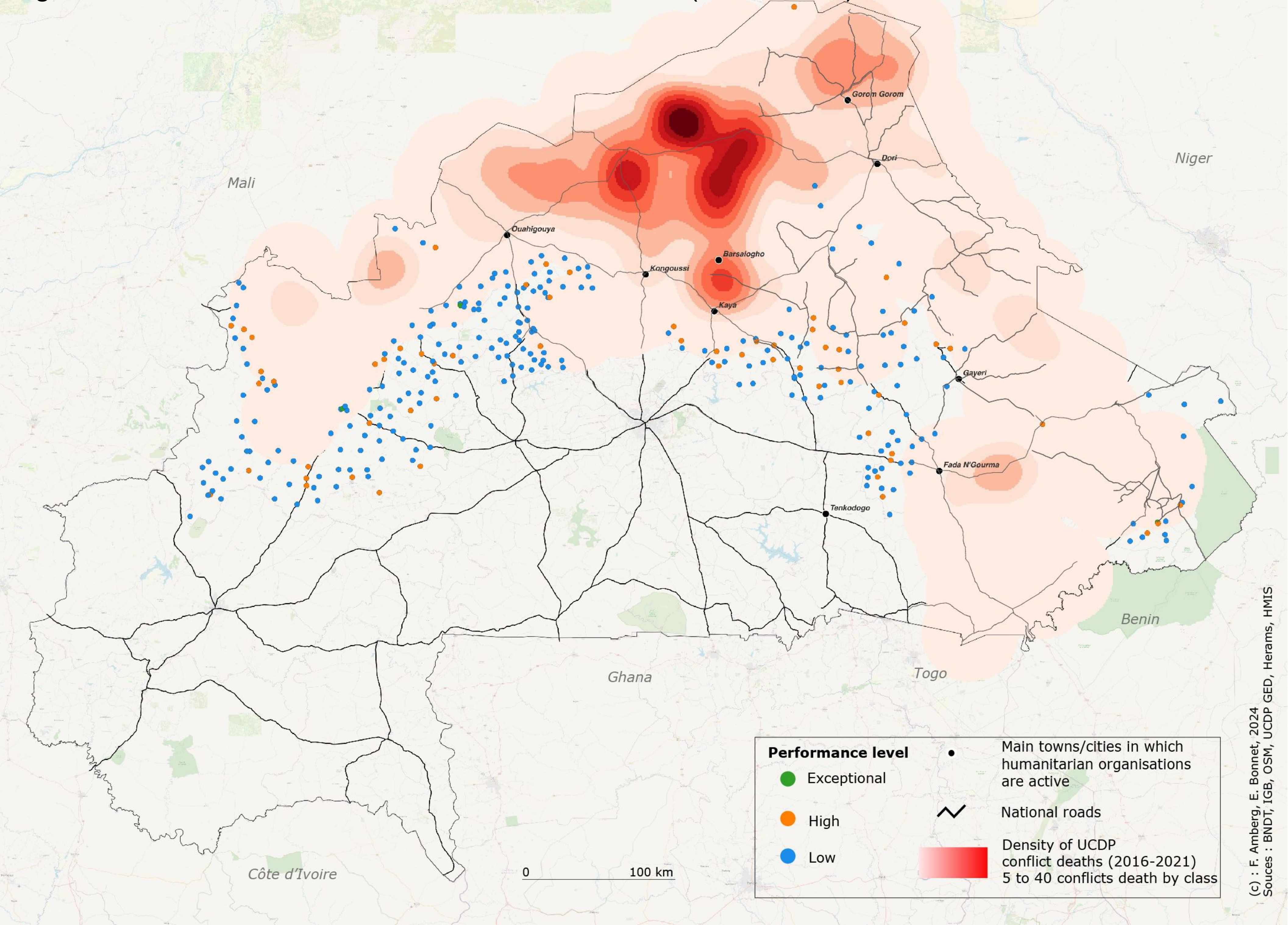

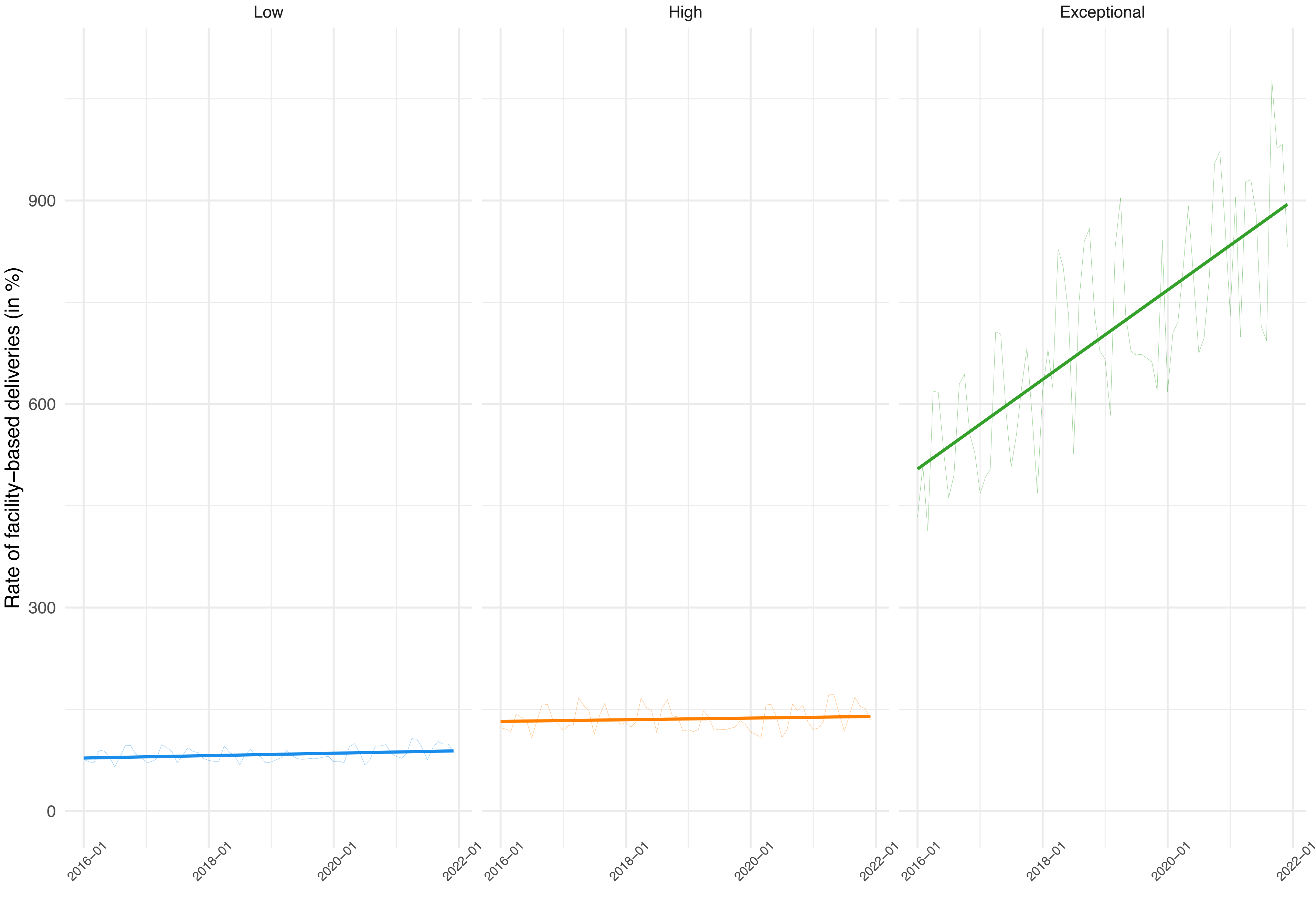

Low
High
Exceptional
Rate of facility–based deliveries (in %)
900
600
300
0
2016–01
2018–01
2020–01
2022–01

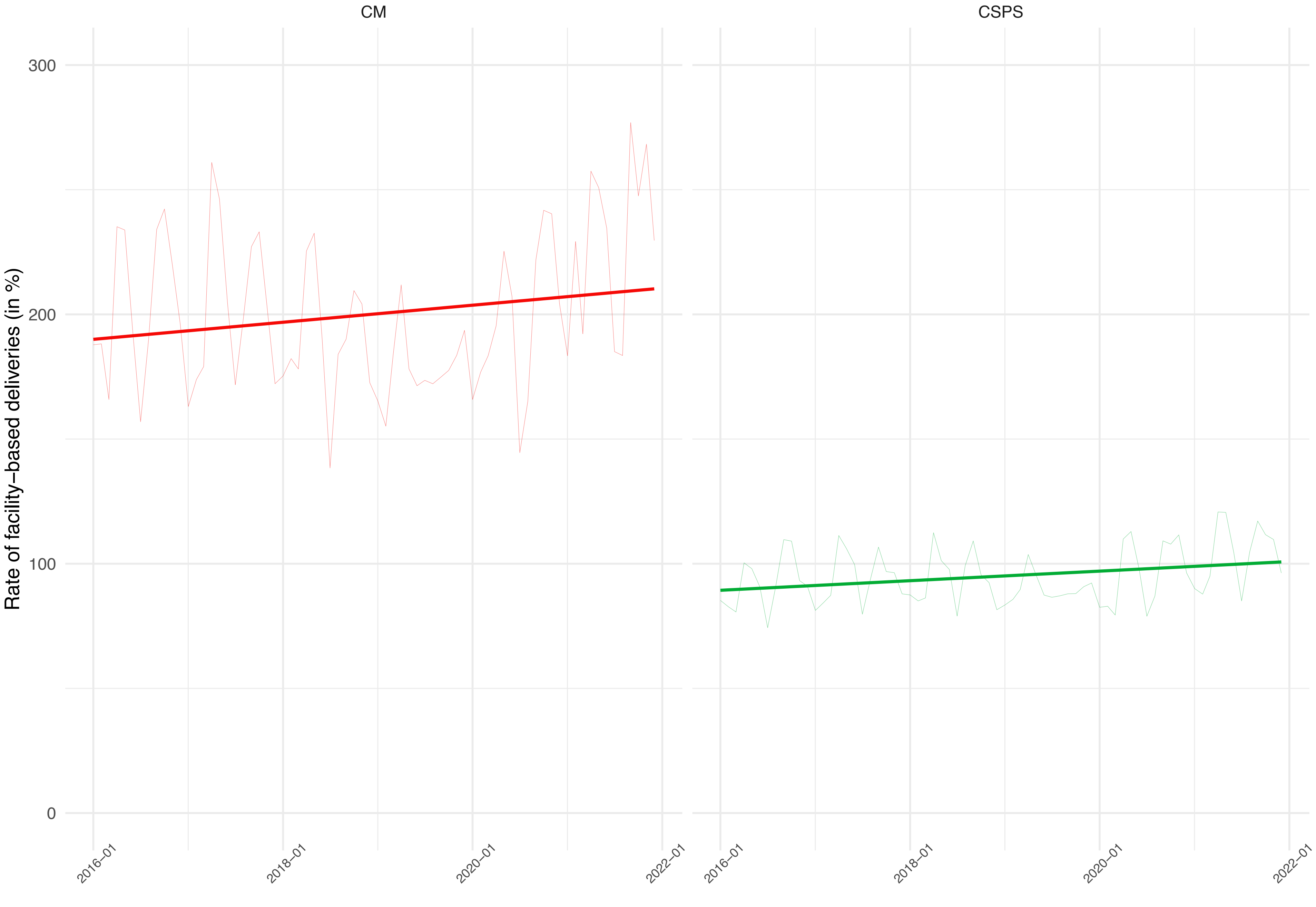
CM
CSPS
Rate of facility–based deliveries (in %)
300
200
100
0
2016–01
2018–01
2020–01
2022–01
